# NetDecoder: a network biology platform that decodes context-specific biological networks and gene activities

**DOI:** 10.1093/nar/gkw166

**Published:** 2016-03-14

**Authors:** Edroaldo Lummertz da Rocha, Choong Yong Ung, Cordelia D. McGehee, Cristina Correia, Hu Li

**Affiliations:** Department of Molecular Pharmacology and Experimental Therapeutics, Center for Individualized Medicine, Mayo Clinic College of Medicine, Rochester, MN 55905, USA

## Abstract

The sequential chain of interactions altering the binary state of a biomolecule represents the ‘information flow’ within a cellular network that determines phenotypic properties. Given the lack of computational tools to dissect context-dependent networks and gene activities, we developed NetDecoder, a network biology platform that models context-dependent information flows using pairwise phenotypic comparative analyses of protein–protein interactions. Using breast cancer, dyslipidemia and Alzheimer's disease as case studies, we demonstrate NetDecoder dissects subnetworks to identify key players significantly impacting cell behaviour specific to a given disease context. We further show genes residing in disease-specific subnetworks are enriched in disease-related signalling pathways and information flow profiles, which drive the resulting disease phenotypes. We also devise a novel scoring scheme to quantify key genes—network routers, which influence many genes, key targets, which are influenced by many genes, and high impact genes, which experience a significant change in regulation. We show the robustness of our results against parameter changes. Our network biology platform includes freely available source code (http://www.NetDecoder.org) for researchers to explore genome-wide context-dependent information flow profiles and key genes, given a set of genes of particular interest and transcriptome data. More importantly, NetDecoder will enable researchers to uncover context-dependent drug targets.

## INTRODUCTION

Biological context influences the pleiotropic nature of a gene in shaping diverse biological phenotypes (1,2). The binary on/off, bound/unbound, active/inactive states of molecular constituents represent the ‘information’ encoded in a biological context. The chain of interactions—specifically, protein–protein interactions (PPI) that alter the binary state of a biomolecule—represent the ‘information flow’ within a cellular network (3) that determines phenotypic properties. The functionality of biological processes, such as cell cycle, can be remarkably distinct under different biological contexts, including health and disease (4–6). Understanding the context-specific functionality of biological processes and genes is critical to determining how information flows among different biological states can give rise to diverse biological phenotypes, including various types of diseases.

No computational tool is currently available to dissect context-dependent network and gene activities on a genome-wide scale. Most current pathway and network enrichment analyses (7–9) rely on differentially expressed genes (DEGs) or mutated genes to indicate which biological processes and interaction network modules are statistically over-represented. However, current enrichment approaches do not provide clues on how biological information is conveyed within a context-dependent interaction network. As such, these tools lack the ability to assess the overall functionality of a biological system, which relies upon the sequence of information relays from upstream to downstream signals via a myriad of molecular interactions involving genes that are not necessarily differentially expressed or mutated (10). Given no such method exists to allow researchers to systematically characterize context-specific networks and the respective key genes, it is necessary to develop a quantitative computational approach that can approximate the activity of information relays, dissect subnetworks that are actively engaged in context-dependent activities and quantify the contribution of key genes that are important in ‘re-routing’ context-dependent information flows. The broad biological impact of such an approach is evident: improved understanding of disease aetiology, pathological properties and drug design based on biological contexts.

To address this challenge, we developed NetDecoder, a network biology platform that is capable of reconstructing context-specific network profiles and determining context-dependent information flow profiles using pairwise phenotypic comparative analyses. Our method is inspired by the fact that interactions between proteins are well conserved (11,12), the architecture of the PPI network is modularized by similar or related biological functions under evolutionary pressure (13), and that any two interacting proteins might cooperate in related biological processes. Based on these principles, we designed a process-guided flow algorithm to identify molecular interaction paths that connect a source gene (where information flow begins) to a target gene (also called sink, where information flow ends) with shared biological processes. In so doing, we provide the approximate context-specific information flows of a biological network.

In order to illustrate the utility of NetDecoder in dissecting context-specific subnetworks and key genes that recapitulate biological properties in distinct phenotypes, we obtained transcriptome data associated with breast cancer (ER-positive and ER-negative), dyslipidemia (homozygote and heterozygote) and Alzheimer's disease (incipient, moderate and severe states) as case studies. These three major disease classes represent distinct pathological phenotypes: uncontrolled cell proliferation and malignancy (breast cancer), metabolic syndrome (dyslipidemia) and neurodegenerative disorders (Alzheimer's disease).

Since DEGs directly impact disease phenotype and transcriptional regulators affect gene expression, these genes are used as sources and target genes, respectively. We aim to uncover key ‘intermediary genes’ that modulate context-specific information flows between source and target genes. Although many of these intermediary genes are not differentially expressed, they play important roles in ‘connecting the dots’ (10) and can determine information flow profiles by re-routing information flow paths that give rise to distinct biological phenotypes.

We demonstrate NetDecoder is capable of dissecting subnetworks and the corresponding key players that are specific to a given disease context. We further show that genes residing in disease-specific subnetworks are enriched in disease-related signalling pathways and information flow profiles, which drive the resulting disease phenotypes. We also devise a novel scoring scheme to quantify key genes whose information flow profiles greatly impact disease phenotypes. Lastly, we show the robustness of our results against parameter changes. Thus, we offer NetDecoder as a network biology platform for researchers to explore genome-wide context-dependent information flow profiles and key genes for any set of genes of interest. For the first time, researchers will be able to query context-dependent functionalities of networks and genes, starting from a set of genes of particular interest and transcriptome data. Furthermore, NetDecoder also allows researchers to prioritize drug targets for genes that affect pathological contexts. NetDecoder source code and other materials are available at the website portal http://www.NetDecoder.org.

## MATERIALS AND METHODS

### Gene expression datasets

Publicly available microarray gene expression profiles for breast cancer, dyslipidemia and Alzheimer's disease were downloaded from the GEO database under the accession numbers GSE42568, GSE6054 and GSE28146, respectively. Raw expression profiles were background corrected and summarized into probeset values. Probesets mapping to the same gene were averaged and each array was normalized by dividing each expression value by the total expression value in the respective array as described in our previous work (14).

### Protein interaction database

We downloaded the iRefIndex version 14.0 and constructed an interaction network using all interactions available including direct interaction, physical association, colocalization, association, covalent binding, methylation reaction, phosphorylation reaction, cleavage reaction, genetic interaction, dephosphorylation reaction, ubiquitination reaction, hydroxylation reaction, self-interaction, protein cleavage, acetylation reaction, deubiquitination reaction, ADP ribosylation reaction, deacetylation reaction, enzymatic reaction, palmitoylation reaction, sumoylation reaction, disulfide bond, RNA cleavage, DNA strand elongation, oxidoreductase activity electron, transfer reaction gtpase, reaction, neddylation reaction, transglutamination reaction, demethylation reaction, isomerase reaction, proline isomerization reaction and phosphotransfer reaction. We removed self-loops and multiple edges creating a final iRefIndex index network containing 15 608 proteins and 180 044 interactions. Our network is available for download as an .R object from our website.

### Context-specific weighted interactome

We created a context-specific interactome by integrating a PPI network with a co-expression network generated from context-specific transcriptome data. Briefly, we computed Pearson correlation coefficients (PCC) across samples of the same context (or phenotype such as control and disease) for each interaction pair available in the PPI network. The absolute value of the PCC was used to weight each interaction in the PPI network. These weights were used to define the capacity and cost associated with each interactome edge, as described in the next sections.

### The process-guided flow algorithm

In the process-guided flow algorithm, flow of information via physical PPI goes from source nodes to target nodes through the graph edges with defined capacity and cost that determine the maximum amount of flow an edge is able to carry. By default, genes involved in transcriptional regulation are defined as targets (sinks). Sources may be defined as DEGs, mutated genes, drug targets or any other gene of interest. This flow is analogous to a current flow finding the path with lowest resistance in an electrical circuit. Therefore, the process-guided flow algorithm is formulated as a minimum-cost flow optimization problem where the edge capacities are defined as the absolute value of the PCC and the cost as -log(PCC). We used the PCC to capture phenotype-specific information as co-expression is a good indicator of genes cooperating in the same or related pathways.

The process-guided flow algorithm takes a weighted interactome G(V, E) as input, a list of proteins ${P \subset V}$ to be used as sources, a list of proteins to be used as targets ${T \subset V}$ and applies the following modifications to solve the minimum-cost optimization problem for an arbitrary number of sources and targets:

1. ${V^\prime = V \cup \{ {s,t} \}}$, where *s* and *t* are auxiliary nodes used to transform a single-source single-sink problem into a multiple-source multiple-sink problem.
2. ${E^\prime = E \cup {{( {s,i} )}_{\forall i \in P}} \cup {{( {t,i} )}_{\forall i \in T}}}$, connecting sources and sinks to the auxiliary nodes *s* and *t*.

Each edge is defined by a capacity and cost. Flow is not allowed to be negative or higher than the capacity and each node has to satisfy the local equilibrium condition, which means that the inflow must be equal to the outflow at every node, except at the source and sink. Formally, we define ${{f_{ij}}}$ as the flow from node *i* to node *j* subject to the following constraints:

\begin{equation*}{\sum\limits_{j \in V^\prime} {{f_{ij}}} - \sum\limits_{j \in V^\prime} {{f_{ji}}} = {\rm{0}}\forall i \in V^\prime - \left\{ {s,t} \right\}}\end{equation*}

where $i$ is the node in the PPI network and $V$ is the set of nodes within the PPI network.

\begin{equation*}{\sum\limits_{i \in P} {{f_{si}}} - \sum\limits_{i \in T} {{f_{it}}} = {\rm{0}}}\end{equation*}

\begin{equation*}{{\rm{0}} \le {f_{ij}} \le {c_{ij}}\forall \left( {i,j} \right) \in E^\prime}\end{equation*}

where ${c_{ij}}$denotes capacity from node $i$to node$j$.

The solution is a sparse subnetwork, connecting sources to targets through the interactome edges. Although the solution of the minimum-cost optimization problem is a directed subnetwork, such directionality does not represent causality but only reflects that way the algorithm finds paths connecting sources to sinks. Typically, NetDecoder provides as one of its outputs two sparse subnetworks connecting the input gene set to the transcription-related sinks for any two phenotypes under consideration.

The process-guided flow algorithm uses only one parameter—the size of the functional neighbourhood (SFN) associated with a particular gene—to find paths between sources and targets/sinks. We define a functional neighbourhood as the biological processes (in GO term) associated with the interacting partners of a gene whose edge capacities (i.e. flow capacities that pass through interacting protein pairs) are higher than a threshold (SFN = 0.95 in all calculations presented here). By increasing SFN value, the parameter becomes more stringent, thus always decreasing the number of paths. This is because SFN controls the number of first-neighbours (directly interacting partners) of a gene. The functional neighbourhood is defined using the GO terms of the first-neighbours of a given protein, including the GO terms for the gene under consideration. In this study, SFN = 0.95 is used as a default parameter to allow users to select the most highly correlated interacting partners for a gene under consideration without being too stringent and potentially losing biologically relevant interacting paths. For examples, at higher SFN values such as SFN = 0.99, fewer paths are included and many biologically meaningful paths are excluded. However, users can reduce the SFN values below default value (SFN = 0.95) in order to retrieve more paths that they think might be biologically relevant. The optimal value for SFN is therefore dependent on the user's study goals, although we recommend the default value (SFN = 0.95) is sufficient to include biologically relevant paths in most scenarios.

If there is a shared GO term between two interacting proteins, say protein 1 and protein 2, the shared GO term will be included in the flow paths. If there is no shared GO term, GO terms of their highly correlated interacting partners (with high edge capacities) will be used. Supplementary Figure S17 illustrates how the algorithm decides which path will maximize the flow. The algorithm takes into account the overlap between the functional neighbourhoods of protein 1 and protein 2, considering such overlap as part of the protein 1 functional neighbourhood (GO1, GO2 and GO7). Then, if the functional neighbourhood of protein P1 shares any biological process with the functional neighbourhood of protein 2 (GO1, GO3, GO4 and GO7), protein 2 will be included in the path. When protein 4 is considered as a candidate protein, there is no biological process being shared with the functional neighbourhood of protein 1, therefore no path will go through protein 4 from protein 1.

### Identification of gene signatures (sources)

We used a template-matching approach (15) to identify genes preferentially expressed in disease as compared to control samples. We selected the top 0.5% of genes with the highest template-matching scores, obtaining gene lists with 101 genes for all disease studied in the present work. These gene lists were then defined as sources and used as input for NetDecoder.

### Targets (sinks)

In network flow terminology, targets (sinks) refer to the nodes where flows end. In current study, by default we defined targets as genes involved in transcriptional regulation from GO annotations. We used genes defined under the following three GO terms to establish the targets: GO:0003676 (nucleic acid binding), GO:0006355 (regulation of transcription, DNA-templated) and GO:0008134 (transcription factor binding). However, our algorithm is flexible allowing users to define their own targets (sinks), including genes involved in cell cycle, apoptosis and known drug targets, depending on the their research questions.

### Prioritization of protein interaction pairs

ER-negative breast cancer is used as an illustrative example of how NetDecoder prioritizes protein interaction pairs. To find interactions that might be important in ER-negative breast cancer, we first require that protein interaction pairs (edges) in the ER-negative subnetwork have a flow higher than 0.5 (parameter 1). Then, we computed the ratio of the edge flow values between ER-negative and control subnetworks and require that this ratio is higher than 5 (parameter 2). Next, we performed Principal Component Analysis (PCA) to find the edges that better distinguish the control and ER-negative subnetworks by selecting the top 10 edges with the highest or the top 10 edges with the lowest loadings from each principal component (parameter 3). Same parameter settings were used for all disease cases in this study.

### Jaccard index

To assess overall similarity between phenotype 1 and phenotype 2, edge-centred subnetworks (Figure 2C and Supplementary Figures S2C–S7C), we computed the Jaccard index for the genes shared between both subnetworks. The Jaccard index is defined as the proportion of shared nodes between A and B relative to the total number of nodes connected to A or B.

\begin{equation*}{{J_{AB}} = \frac{{|N\left( A \right) \cap N\left( B \right)|}}{{|N\left( A \right) \cup N\left( B \right)|}}}\end{equation*}

We used this metric to define B = A to assess, for example, if gene A in the phenotype 1 subnetwork has the same interacting partners in phenotype 2 subnetwork or has established new interactions as a function of the different transcriptomes associated with phenotypes 1 and 2.

### Network routers

We define network routers as intermediary proteins that have influence over many genes and show high flow differences between two phenotypes. To find network routers, we computed the difference in total flow (the in and out flows) of each gene between phenotype 2 and phenotype 1 subnetworks. As our interactomes are context-specific, it might happen that some genes, edges and paths are unique to a given phenotype. When a gene is present only in one context-specific subnetwork, we define its total flow in the other network as zero to compute the flow differences.

### Key targets

We define key targets as the target/sink nodes (genes related to transcriptional regulation) with high flow differences. The procedure to compute flow differences is similar to the one used to compute network routers with the only difference being that the network router is located at an intermediary location (i.e. between source and target/sink) and the key target is located where flow ends.

### High impact genes

We defined high impact genes as genes that experience a significant change in regulation between control and disease conditions including flow differences, establishment of new inflows and change of directionality of gene expression correlations (i.e. from positive correlation to negative correlation or vice versa) between two phenotypes. Thus, unlike network routers and key targets where their locations within the flow path are definite, a high impact gene can be located either at an intermediary or at an ending path location. We developed a novel scoring scheme, termed impact score (IP), to rank and assess genes based on their importance in mediating differences in information flow profiles between two given phenotypes. The impact score for gene *gi* is defined as

\begin{equation*}{{\rm I}{{\rm P}_{gi}} = \left( {{\rm TF}g{i_{{\rm phenotype}{\rm{2}}}} - {\rm TF}g{i_{{\rm phenotype}{\rm{1}}}}} \right) * {\rm NIE} * {\rm NCD}}\end{equation*}

where ${{\rm TF}g{i_{{\rm phenotype}{\rm{2}}}}}$ is the total flow on *gi* in the phenotype 2 subnetwork, ${{\rm TF}g{i_{{\rm phenotype}{\rm{1}}}}}$ is the total flow of *gi* in phenotype 1 subnetwork and NIE is the number of new inflow interactions established by *gi* in the phenotype 2 subnetwork normalized by the sum of the total number of interactions established by *gi* in phenotype 1 and phenotype 2 subnetworks. NCD is the number of gene expression correlations that changed directionality (i.e. from positive correlation to negative correlation or vice versa) between phenotype 1 and phenotype 2, which is independent of the magnitude of gene expression correlations with the same directionality. We define genes with high IP scores as high impact genes. Importantly, the IP score is defined only for genes that occur in both subnetworks of phenotype 1 (control) and phenotype 2 (disease). In some instances, high impact genes could also be network routers or key targets.

### Computational complexity

To assess computational complexity, we used the Ford–Fulkerson algorithm (16) to find the maximum flow through the flow network. In a maximum-flow problem (no optimization to find low cost paths), the Ford–Fulkerson implementation based on a breadth-first search has a performance of O(V*E^2^). To find the lowest cost paths, we used the PRIM's algorithm (17) whose performance when implemented using a binary heap is O((V+E)*logV). Here, V is the number of vertices and E is the number of edges in the network, respectively.

### Pathway enrichment analysis

We used NIH DAVID to perform enrichment analysis of genes contained in context-specific subnetworks for canonical pathways in KEGG database that are significantly enriched (Benjamini-corrected *P*-value < 0.05).

## Source code

The source code of NetDecoder is freely available at Bitbucket and www.NetDecoder.org. An online website portal (www.NetDecoder.org) was built to include the comprehensive step-by-step tutorial to guide the users to download, install the source code, run the software locally and better use the software to interpret NetDecoder results. An online forum group is also available under Google Groups (https://groups.google.com/forum/#!forum/netdecoder) to report bugs, troubleshot problems or discuss the software online.

## RESULTS

### Acquiring context-specific subnetworks and key genes via process-guided flow algorithm

**Figure 1.**
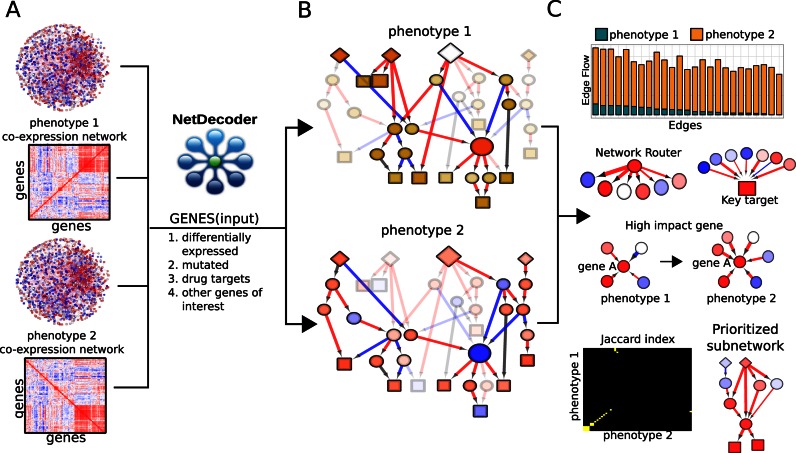
Schematic overview of process-guided flow algorithm in NetDecoder platform. (**A**) Using the global transcriptome for phenotype 1 (control) and phenotype 2 (disease), we computed the PCC for each protein interaction pair (edge) reported in the protein interaction network to devise a gene co-expression network for each phenotype (context). The PCC matrix, illustrated as a heatmap, is then used to derive an edge-weighted interactome for each phenotype (i.e. each edge is weighted based on the PCC value of a given phenotype), which is used as input for NetDecoder. (**B**) A gene list, such as differentially expressed or mutated genes, drug targets or any other gene list of interest is also required as input to NetDecoder. These genes are called sources. Both sources (diamonds) and sinks or target nodes (squares) can be defined based on user's study goal. By default, genes involved in transcriptional regulation are targets. The process-guided flow algorithm is then used to select paths along the phenotype-specific edge-weighted PPI networks. These paths start at the source nodes (diamonds) and end at the target nodes (squares), passing through intermediary nodes (circles). By using the same genes as sources but with a phenotype-specific interactome, NetDecoder can find different phenotype-specific paths. Red nodes are genes with high flow difference between phenotype 2 and phenotype 1 and vice versa for blue nodes. Red edges are protein interaction pairs positively correlated while blue edges are negatively correlated. (**C**) Next, edges that show distinct edge flow across protein pairs (edges) in two phenotypic states are identified. Then, the following key genes within the phenotype-specific subnetworks are identified: (i) network routers, (ii) key targets and (iii) high impact genes. Network routers are intermediary proteins that show high flow differences (in- and outflows) between two phenotypes, key targets are target (or sink) nodes that show high flow differences between two phenotypes, and high impact genes are genes that show extensive alterations in flows between two phenotypes, including flow differences, establishment of new inflows and directionality changes in gene expression correlations. The overall similarity between subnetworks is evaluated with the Jaccard index and prioritized subnetworks are generated by comparisons between phenotype 1 and phenotype 2 subnetworks.

NetDecoder is designed to perform any pairwise phenotypic comparison that interests a researcher. For a phenotypic pair, such as phenotype 1 and phenotype 2 (Figure 1A), we always obtained two subnetworks, one corresponding to phenotype 1 versus phenotype 2 and the other one corresponding to phenotype 2 versus phenotype 1 (Figure 1B). Both subnetworks capture different phenotypic aspects, depending on a user's research interest. In the current study, we compare health (control) phenotype and disease phenotype and obtain two subnetworks, one corresponding to disease versus health and the other health versus disease. As will be seen in key gene motifs discussed below, comparing flow properties of health and disease subnetworks illuminates insight into disease aetiology and sheds light on how to design context-specific therapeutics to ‘reverse’ a disease phenotype.

We sought to find network paths (i.e. a sequence of protein interaction pairs from source to target nodes) that are enriched with highly correlated PPI for a given disease context (Figure 1B). In our study, source nodes are genes whose differential expression is observed in a specific phenotype and target nodes are genes whose functions are related to transcriptional regulation. We developed a process-guided flow algorithm to model information flows from source to target nodes through a protein interaction pair (edge). We first computed PCC and defined the weights (i.e. capacity) associated with each edge as the absolute value of PCC (Figure 1A). The extent of information flow at each edge is thus correlated to PCC values (both positive and negative correlation coefficients) and flows are allowed when interacting protein pairs share biological processes. Thus, given a set of source genes, NetDecoder is able to generate context-specific subnetworks corresponding to any pairwise phenotypic comparison (Figure 1B).

Further characterization of context-specific subnetworks reveals that there are three distinct types of network properties from a signal flow perspective: (i) flow differences at intermediary locations (network routers), (ii) flow differences at ending locations (key targets) and (iii) alteration of flow paths and directionality of gene expression correlations (high impact genes) (Figure 1C). Here, network routers are intermediary genes with high flow differences when comparing two context-specific subnetworks. Key targets are sinks (genes related to transcriptional regulation) where flows end with high flow difference between two phenotypes under comparison. High impact genes are genes whose flow profiles show profound changes between two phenotypes that include establishment of new flow paths and gene expression correlation changes of directionality from positive to negative or vice versa. Both network routers and key targets capture flow differences between two phenotypes, with network routers emphasize on in- and outflow differences at intermediary locations and key targets on total incoming flows where flows end. Network routers exhibiting high flow differences indicate their influence over specific flow paths in a given biological context. Key targets exhibiting high flow difference act as key regulators to modulate downstream responses that help to maintain a given phenotype. High impact genes capturing the establishment of new flow paths and directionality changes of gene expression correlations are critical to understand rewiring events that lead to differential signal flows between two phenotypes. The network properties of network routers, key targets and high impact genes are illustrated in Figure 1C.

### Characteristics of ER-negative-specific edge-centred subnetwork

**Figure 2.**
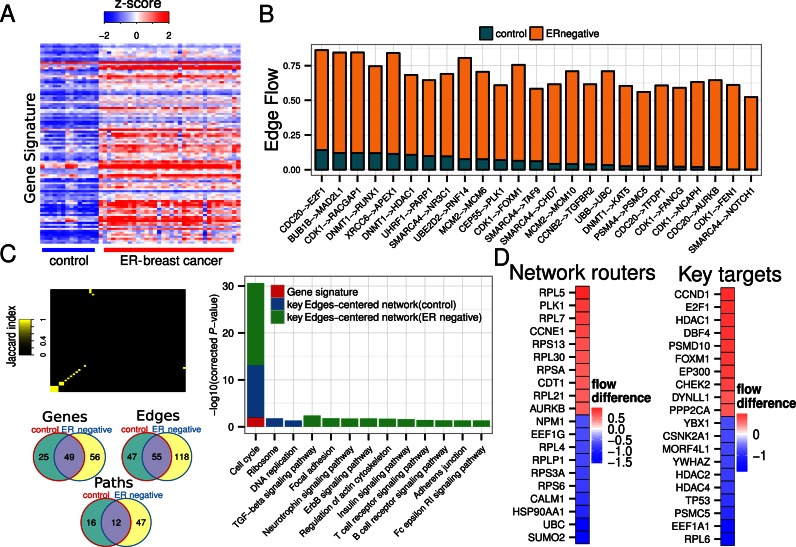
Properties of ER-negative breast cancer-specific edge-centred subnetwork. (**A**) Heatmap of DEGs across control and ER-negative breast cancer. Z-score transformation was applied for visualization purposes. DEGs are used as source nodes to identify disease-specific subnetworks (red: positive z-score; blue: negative z-score). (**B**) Total edge flow profiles in control and ER-negative breast cancer for edges with higher flows in disease than in control subnetworks. (**C**) Jaccard index to evaluate the similarity between control-specific and ER-negative breast cancer-specific subnetworks and Venn diagrams showing the overlap across genes, edges and paths. Pathway enrichment analysis for (i) gene signatures (DEGs) (red), (ii) key edge-centred subnetworks in control (blue) and (iii) disease (green). Pathway enrichment analysis for genes that compose disease-specific, edge-centred subnetworks captures signalling pathways that are relevant to disease aetiology. (**D**) Heatmap showing node flow differences across control and ER-negative breast cancer for top 20 network routers and key target genes showing high node flow difference (red) and low node flow difference (blue) in ER-negative breast cancer.

To demonstrate how NetDecoder illuminates the paths between DEGs (source) and transcriptional regulators (sink) that drive disease pathogenesis, we examined ER-negative breast cancer as an illustrative example. With ER-negative breast cancer DEGs as sources (Figure 2A) and transcriptional regulators as targets, networks specific to corresponding health (control) cases and ER-negative breast cancer were obtained. We performed a PCA to identify protein interaction pairs (edges) that show distinct edge flow in ER-negative breast cancer (Figure 2B). The resulting ER-negative breast cancer-specific edge-centred subnetworks or subnetworks enriched with protein interaction edges of high edge flows (Supplementary Figure S1) captures (i) total flow difference of each gene in the disease state as compared to the control state; (ii) co-expression correlation of each interaction pair and (iii) total edge flow of each interaction pair. The function of two proteins is deemed to be highly coordinated when an interacting protein pair shows positive gene co-expression correlation with high flow difference. Analysing coordinated or uncoordinated functions for key genes (network routers, key targets and high impact genes) in edge-centred subnetworks can illuminate how these genes drive a disease phenotype.

Analyzing edges in an ER-negative breast cancer-specific, edge-centred subnetwork indeed reveals a number of genes such as *CDC20, CDK1* and *E2F1*, which are known to be involved in tumourigenesis. Interestingly, ER-negative-specific edges also captured genes relevant to cancer prognosis. *SMARCA4* (18), *RUNX1* (19) and *FOXM1* (20) have been reported to contribute to poor prognosis in breast cancer patients. We not only detect *FOXM1* in our ER-negative-specific, edge-centred subnetwork but also we further identify an edge that supports *FOXM1* and *CDK1* interaction in disease (Figure 2B). The role of these two genes in cell cycle regulation is well documented in the literature.

NetDecoder further captures two types of key genes in terms of flow differences: network routers and key targets. Figure 2D illustrates the top 10 network routers and key targets with the most positive and negative flow differences between ER-negative breast cancer and control cases, respectively. Network routers are located at intermediary flow path locations (i.e. between sources and targets/sinks) and key targets are located where flow ends. High positive flows in network routers indicate more ‘intensive’ information flows being mediated by these genes; in disease states, network routers mediate ‘re-routed’ preferential information flows. In contrast, negative flows through network routers indicate low information flow suggesting ‘unfavourable’ information flow through these genes in the disease state. The same scenario also applied for key targets except that instead of re-routing information flows as for the network router, key targets are genes where information converged, with high positive flows indicating the higher importance of modulating transcriptional events in the disease state.

**Figure 3.**
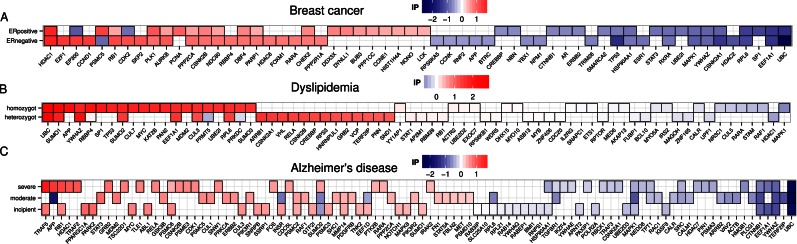
High impact genes. Heatmaps for top 40 genes with high impact scores in (**A**) breast cancer, (**B**) dyslipidemia and (**C**) Alzheimer's disease. The impact score (IP) ranks genes based on node flow differences between control and disease-subnetworks, the proportion of newly established interactions in the disease subnetwork, and also the number of edges whose expression correlation changed directionality (for example, an interaction that is negatively correlated in control becomes positively correlated in the disease subnetwork). The more extreme the IP score (positive or negative) the more likely it is a gene that contributes to disease aetiology.

In addition, NetDecoder also identified *FOXM1* as a top scored gene using two different scoring metrics: *FOXM1* is both a key target (Figure 2D) and a high impact gene (Figure 3A) further suggesting the importance of *FOXM1* in ER-negative breast cancer pathogenesis. NetDecoder thus captures two additional known prognostic markers, *SMARCA4* and *RUNX1*, that were not reported by the original study that aimed to uncover prognostic markers using the same gene expression dataset (20).

To show that subnetworks generated by NetDecoder are indeed context-specific, we examined the similarity of the corresponding control and ER-negative breast cancer edge-centred subnetworks using the Jaccard index (21), which measures the extent of interacting partners shared by the same gene in these two subnetworks. The resulting heatmap globally shows that although some genes have the same or similar interacting partners, the majority show distinct interactions (Figure 2C). Venn diagrams for ER-negative and control edge-centred subnetworks show that although these subnetworks have some common genes, edges and paths, there are substantial differences between both subnetworks (Figure 2C). We therefore demonstrate that NetDecoder can generate context-specific subnetworks given the same set of source and target genes.

We next performed pathway enrichment analyses using both gene signature (DEGs) and genes residing in control and ER-negative breast cancer edge-centred subnetworks to determine whether the profiles of these genes capture biological signalling pathways that signify disease properties. Our analyses revealed a number of enriched canonical signalling pathways such as cell cycle, TGFβ signalling and focal adhesion, which when dysfunctional are cancer hallmarks (22) (Figure 2C). Cell cycle appears as the most enriched process that is also captured by gene signature. However, other disease-relevant pathways are not enriched using gene signature (DEGs) alone indicating gene signature is not sensitive enough to capture disease-relevant signalling pathways. Interestingly, a number of these disease-associated signalling pathways are not enriched by genes obtained from control-specific edge-centred subnetworks. The exception is cell cycle pathways enriched in both control cases and disease cases that exhibit remarkably distinct functional behaviour, as discussed in Supplementary Discussion. Our results indicate edge-centred subnetworks derived from ER-negative breast cancer recapitulate pathways that contribute to the malignant and proliferative characteristics of breast cancer that are distinct from controls. Results of similar analyses for other diseases are also provided in Supplementary Figures S2–S7.

Further examination of ER-negative breast cancer-specific edge-centred subnetworks indeed reveals genes and paths capturing major processes that can explain disease behaviour (Supplementary Figure S1). The path NRAS-BRAF-MAP2K2-MAPK1 from the canonical MAPK cascade, which is important in tumourigenesis, is captured in the ER-negative breast cancer-specific edge-centred subnetwork. In addition, this subnetwork also captures key transcriptional regulators in tumourigenesis such as RB1, E2F1, FOXM1 and HDAC1. Interestingly, the cyclin-dependent kinase CDK1 shows low values of flow difference between disease and control cases but exhibits positive correlations and large edge flows with genes acting as cell cycle regulators, indicating its information flow is more directed towards these cell cycle regulators in promoting cell proliferation in the disease context.

### Information flow-mediated by key genes in disease-specific subnetworks reveals disease aetiology

**Figure 4.**
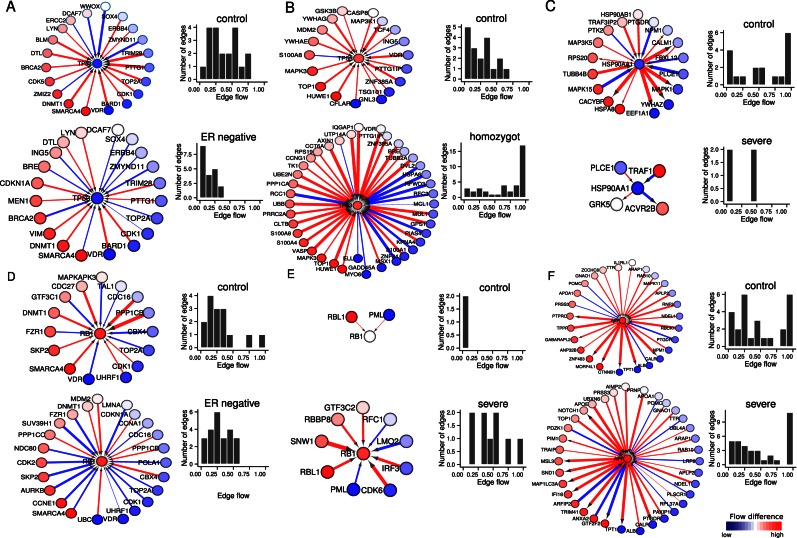
Context-specific profiles of disease key gene motifs. (**A**) TP53 is a key target gene for ER negative breast cancer. (**B**) TP53 is a high impact gene in dyslipidemia homozygote. (**C**) HSP90AA1 is a network router in AD severe. (**D**) RB1 is a high impact gene for ER negative breast cancer and (**E**) AD severe. (**F**) APP, a high impact gene in AD severe. Each gene node is coloured according to the node flow differences across control and disease as displayed by the gradient bar at the top left. Edge thickness represents the amount of edge flow. The edge flow direction is determined by the definition of source and target nodes with a path starting at the source and ending at target nodes. Red edges indicate a positive gene expression correlation between a pair of protein interactions, while blue edges represent a negative correlation. The edge flow distribution for each gene is shown in the bar chart at the right.

Representative motifs of disease-specific key genes are shown in Figure 4. We captured tumour suppressor gene TP53, which helps to maintain the integrity of a genome (23), as a key target in ER-negative breast cancer (Figures 2D and 4A) and a high impact gene in dyslipidemia homozygote (Figure 3B). Interestingly, TP53 shows much reduced overall edge flow in ER-negative breast cancer (bar chart in Figure 4A). In general, there are uncoordinated functions (negative co-expression correlations) between TP53 and DNA damage response gene BRCA2, cyclin-dependent kinase 1 (CDK1), scaffold protein DCAF7, oncogene ERBB4, chromatin remodeller SMARCA4, transcriptional regulator SOX4 and DNA topoisomerase TOP2A in ER-negative breast cancer (Figure 4A). It is also interesting to note that there is coordinated function (positive co-expression correlation) between cyclin-dependent kinase inhibitor 1A (CDKN1A) with TP53. Our results suggest ‘uncoupled’ functional correlation between TP53 and positive cell cycle regulators and DNA damage response genes (Figure 4A), leading to uncontrolled cell proliferation in ER-negative breast cancer.

In contrast, the edge flow profile of TP53 in dyslipidemia homozygote is quite different from ER-negative breast cancer (Figure 4B). In the control case, TP53 shows functional coordination with its negative regulator MDM2 and metabolic regulator glycogen synthase kinase GSK3B, indicating regulated TP53 activity and metabolism. In dyslipidemia homozygote, cell adhesion modulator clathrin CLTB, DNA damage response proteins GADD45A and AXIN1 show coordinated roles with TP53, indicating involvement of DNA damage and altered cellular morphology in the pathogenesis of dyslipidemia. However, unlike cancer, the function of proteins involved in DNA damage response, such as BRCA2 and TOP2A, does not show uncoordinated functions.

Interestingly, tumour suppressor gene *RB1* (retinoblastoma 1) is a high impact gene in ER-negative breast cancer (Figure 3A) and Alzheimer's disease (AD) severe (Figure 3C). We sought to characterize how RB1 can provide clues to understanding the pathological properties of these two diseases that seem to show distinct behaviours (with highly proliferative properties in cancer and degenerative properties in AD). In general, RB1 shows uncoordinated function with positive cell cycle regulators such as CCNA1, CCNE1, CDK1, CDK2, DNA polymerase POLA1 and chromatin remodeller SMARCA4 in ER-negative breast cancer, but shows functional coordination with MDM2 (a negative regulator of TP53) in ER-negative breast cancer (Figure 4D). As with TP53, the overall edge flow for RB1 also decreased in cancer (bar chart in Figure 4D), suggesting loss of tumour suppressing activities of RB1 leading to uncontrolled cell proliferation in cancer.

However, RB1 shows much higher overall edge flow in the AD severe case, which is quite different from ER-negative breast cancer (bar chart of Figure 4E). Instead of directing flows with cell cycle regulators as in the case of breast cancer, there are in general uncoordinated functions between RB1 with transcriptional regulators LMO2 and PML and RFC1 that regulate DNA replication. On the other hand, genes such as retinoblastoma-like RBL1 and transcriptional regulators SNW1 and GTF3C2 show functional coordination with RB1 where their pathological basis in AD remains to be elucidated.

Given extensive extracellular β-amyloid (Aβ) plaques and intracellular neurofibrillary tangles are hallmarks of AD patient brains (24,25), we sought to investigate the role of the heat shock protein HSP90AA1 and amyloid precursor protein (APP) in severe AD. We found HSP90AA1, which acts as a molecular chaperone to promote protein folding, is a network router in our model of an AD severe case (Supplementary Figure S7D). As shown in Figure 4C (bar chart), the overall edge flow in HSP90AA1 decreases markedly in the AD severe case, consistent with clinical observations that misfolded protein aggregates are abundant in the brain of AD patients. Intriguingly, APP, which is known to go awry in AD, is among the top, high impact genes in severe AD (Figure 3C). In addition, the prion protein (PRNP) shows positive correlation with APP in the AD severe case (Figure 4F), thus highlighting preferential functional coordination of these two proteins in AD pathogenesis.

### Prioritized subnetworks provide clues for biological processes that establish context-specific disease states

**Figure 5.**
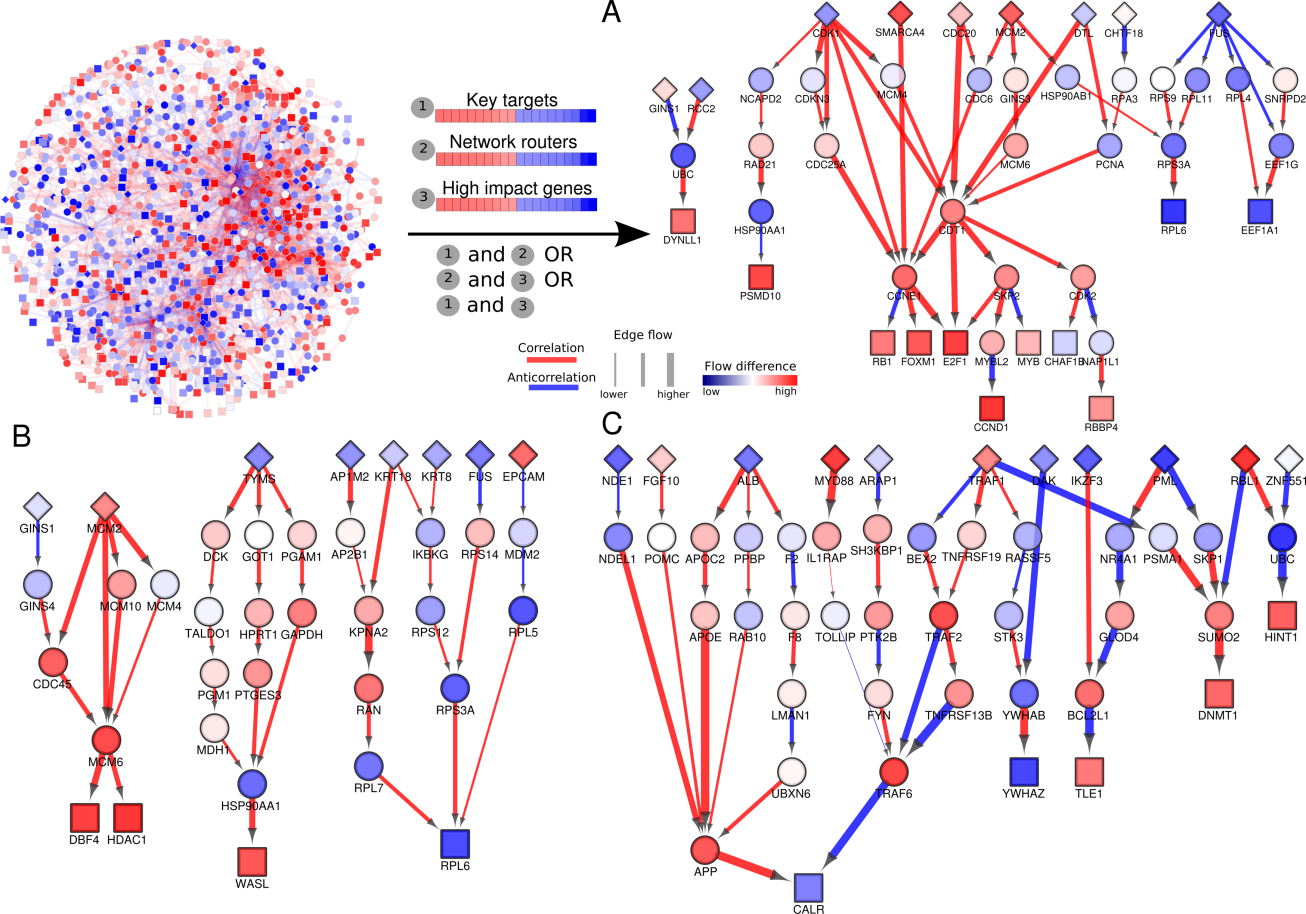
Prioritized disease-specific subnetworks. For a path to be prioritized within a disease-specific subnetwork, it has to fulfil one of the following criteria: (i) key targets and network routers are included, or (ii) key targets and high impact genes are included or (iii) network routers and high impact genes are included. Prioritized disease paths of (**A**) ER-negative breast cancer, (**B**) ER-positive breast cancer and (**C**) AD severe state. Source genes are indicated as diamonds, intermediary genes as circles and target genes (sinks) as squares and coloured according to the node flow difference (red = high flow in disease but low flow in control, blue = low flow in disease but high flow in control). Edge thickness represents the amount of flow through an edge. Additionally, edges are coloured according to the correlation directionality (red for positive correlation and blue for negative correlation).

To further dissect paths that are relevant in disease phenotypes, we selected paths enriched with at least two types of key genes and termed these paths as prioritized subnetworks (Figure 5). Our results indicate a prioritized subnetwork for ER-negative breast cancer captures paths enriched with cell cycle regulators such as CDK1, CDC20, CDC25A, PCNA and CCND1 (Figure 5A). Key transcriptional regulators that had been implicated in breast cancer pathogenesis such as RB1, FOXM1 and E2F1 are key targets within our model and the high values of flow differences further highlight their importance in breast cancer.

In contrast, cell cycle regulators (CDC20 and CCND1), prognostic markers (FOXM1) and tumour suppressors (RB1) observed in ER-negative breast cancer are not captured in the prioritized ER-positive breast cancer subnetwork (Figure 5B). Such findings are consistent with clinical observations (26), indicating that ER-positive breast cancer is less malignant than ER-negative breast cancer. Paths that contained mini-chromosome maintenance proteins involved in the initiation of genome replication show large values of flow difference, and positive correlation-associated edge flows are observed. The prioritized ER-positive breast cancer subnetwork also shows paths marked by metabolism regulators such as TYMS, GOT1, GAPDH and PGM1. Genetic variants of TYMS (thymidylate synthetase) involved in folate-mediated one-carbon metabolism in DNA synthesis have been recently reported in breast cancers (27). A number of ribosomal proteins also show functional coordination in the prioritized ER-positive breast cancer subnetwork. Our result indicates deregulated protein synthesis plays an important role in the pathogenesis of ER-positive breast cancer.

In contrast to our examination of a cancer subnetworks, the prioritized subnetwork for the AD severe state centres around regulators in cytoskeletal regulation (ALB, APP), lipid transport (APOC2, APOE), immune-related responses (MYD88, TRAF1, TRAF6, TNFRSF19) and protein folding and degradation (CALR, PSMA1, UBC) (Figure 5C). Immune responses and the accumulation of unfolded proteins are known to play key roles in the aetiology of AD (28,29). In addition, deregulated activities of lipid transport proteins such as APOE can modify the transendothelial blood–brain barrier transport of beta-amyloid. Our finding showing high edge flows of APOE and positively correlate to APP expression in AD severe is consistent with the recent finding that there is increased APOE activity in AD brain capillaries (30).

In general, our results show prioritized disease-specific subnetworks highlight key PPI paths that are important in mediating disease-specific information flows via key genes, thereby illuminating key cellular processes and their potential roles in driving disease progression.

### Assessment of NetDecoder robustness

To evaluate the sensitivity of NetDecoder, we varied the values of the single NetDecoder parameter, the SFN. We assigned distinct values to SFN (SFN = 0.91, 0.93, 0.97 and 0.99) and compared the output under these conditions to that obtained under default conditions (SFN = 0.95). Here, an ER-negative breast cancer-specific subnetwork is used as an example, whereby the SFN parameter can be adjusted to increase or decrease the number of paths identified by NetDecoder. Increasing the SFN value (a more stringent criterion) will reduce the SFN associated with a given gene and only include its highly correlated interacting partners. In contrast, decreasing the SFN value (a less stringent criterion) will increase the SFN allowing the identification of an increased number of paths that connect sources to targets, as indicated in Supplementary Figure S16. We show the overlap of paths identified by NetDecoder using SFN = 0.91, 0.93, 0.97 and 0.99 compared to paths found using SFN = 0.95 (default) in Supplementary Figure S16A (top panel). In general, these results suggest that although some paths are specific to a defined cutoff, the overlap between paths is large and most of the phenotype-specific paths are recovered under these conditions. The number of predicted paths detected under a certain SFN is shown in the table at the bottom panel of Supplementary Figure S16A.

We also evaluated how changes in SFN affect high impact genes, network routers, key targets and key edges. We selected the top 40 key genes (top 20 most positive scores and top 20 most negative scores) to assess the effects of different SFN values. The majority of high impact genes (33/40) discovered under the default condition (SFN = 0.95) are properly recovered and the lowest value (28/40) is obtained when SFN = 0.91 is used (Supplementary Figure S16B). We evaluated the sensitivity of NetDecoder to recover key targets and network routers (Supplementary Figure S16C and D), and found most of the key targets and network routers are properly recovered with at most seven genes added or removed from the prioritized set of key targets and 13 genes for the network routers when SFN = 0.99 is used.

In evaluating whether key edges are properly recovered (Supplementary Figure S16E), we found that, despite the changes in SFN, the overlap between edges discovered with SFN = 0.95 and across the cutoffs tested is high, except for SFN = 0.91 where 17 new edges were found and 14 edges were removed (Supplementary Figure S16A). These findings illustrate the paths obtained from default SFN = 0.95 are stable and biologically relevant to a given phenotype and that most of these paths are indeed captured using different parameters (SFNs from 0.91 to 0.99). Taken together, our analyses suggest that the NetDecoder results are robust.

## DISCUSSION

Our proof-of-principle survey of context-dependent biological activities, using transcriptome data derived from three distinct disease classes, is the first of its kind. We developed a NetDecoder platform that implements a process-guided flow algorithm to model context-dependent information flows in biological systems based on the principles that the architecture of PPI networks is evolutionarily constrained by biological functions a protein can play and information flows within a cell are mainly mediated via direct physical interactions. Using DEGs as sources and transcriptional regulators as targets, we are able to dissect subnetworks specific to corresponding control and disease cases in this study. Pairwise comparative analyses of these control and disease subnetworks reveal interaction paths that show ‘extreme’ profiles that are unique to a phenotype (Note: ‘extreme’ refers to the amount of flow at very high and very low levels, by comparing disease and control subnetworks).

Conventional network analyses include network-based pathway enrichment (8,9), which conveys only the static topology of a PPI network. NetDecoder, on the other hand, captures context-dependent properties of a phenotype-specific PPI network in terms of information flows. Unlike the conventional definition of hubs, which refers to network nodes linking a large number of associated partners comprising the topological structure of a network, and driver genes whose functions can drive disease progression or the transition of biological states, our definitions for key genes (network routers, key targets and high impact genes) capture shared properties of hubs and driver genes from an information flow perspective. Large flow differences and significant changes in flow profiles for our defined key genes indeed indicate they are ‘hubs’ (due to large flow difference) and ‘driver genes’ (due to their importance in influencing information flows) from an information flow perspective. Since key targets are nodes where flows end, their flow properties are highly dependent on ‘upstream’ candidates such as network routers and high impact genes. Given network routers are defined as intermediary proteins and key targets are sinks (where flows end) the identities of network routers and key targets never overlap. High impact genes, which capture the establishment of new flow paths and directionality changes of gene expression correlations, can be located at intermediary locations or at sinks within the PPI network; hence, the identity of high impact genes, in principle, can overlap with both network routers and key targets. Thus far in our case studies of breast cancers, dyslipidemia and Alzheimer's disease, we did not find overlap between network routers and high impact genes; however, overlap did exist between high impact genes and key targets.

Pathway enrichment analyses of genes in these context-specific subnetworks further explain their corresponding phenotypic properties. Further characterizations of key gene motifs reveal their distinct flow profiles and thus their biological properties in different biological contexts. To demonstrate how the same key genes may play distinct roles in the aetiology of different diseases, we show the remarkably different, context-specific flow profiles of *TP53* and *RB1*.

In addition, we show how key genes can affect context-dependent properties of common biological processes, including canonical cellular processes (in particular MAPK-mediated cascades), cell cycle, transcription, chromatin remodeling, epigenetic, and genome integrity, unfolded protein responses, protein synthesis, cell–cell adhesion and intracellular trafficking (Supplementary Discussion). These common biological processes (including signalling pathways that are associated with these processes) are commonly enriched in many transcriptome analyses; however, the ability to characterize their context-specific activities has been lacking. We therefore provide a more detailed discussion of the context-dependent activities of these common biological processes involving selected key genes.

We reason that our results—the identities of key genes such as network routers, key targets, high impact genes—cannot be directly extracted from the topology of a PPI network; however, they can be gleaned from an examination of information flow within a context-specific PPI network. To illustrate context-dependency for a given key gene, we computed the flow paths from sources that lead to FOXM1. As indicated in Supplementary Figure S18, FOXM1 is a key gene (high impact gene and key target) in the ER-negative breast cancer context, but it is not a key gene in the ER-positive breast cancer context. The key network routers and high impact genes that contribute flows to FOXM1 in each breast cancer context also differ. FOXM1 is a known prognosis marker in breast cancer, and we show it is the relative gene activities in the transcriptome of ER-negative breast cancer that determine its role in breast cancer pathogenesis and prognosis. Our results thus highlight the importance of cellular context in shaping the roles of key genes in a given phenotype.

Key genes identified by NetDecoder, especially those such as high impact genes that show extensive alterations of flow difference and rewiring events, are potential drug targets; key genes are determined by the transcriptome context and targeting their activities can help to restructure the properties of information flows in disease networks in order to achieve therapeutic effects. For instance, HDAC1 is a high impact gene in ER-negative breast cancer (Figure 3A) and a recent study showed inhibiting HDAC can sensitize cancer cells to radiotherapy (31). In the AD context, increased acetylation of chromatin histones in the hippocampus is associated with enhanced contextual learning (32), while reduced acetylation corresponds with impaired memory (33). We found HDAC1, which removes acetyl groups from the acetylated chromatin causing impaired memory, is a high impact gene in the AD severe context (Figure 3C). A recent study showing that inhibiting the activities of HDACs increased histone acetylation and enhanced hippocampal-dependent memory formation (34) further support our result that HDAC1 indeed plays a pathogenesis role in AD and may be a potential therapeutic target. In addition, prioritized disease networks identified by NetDecoder capture context-dependent processes that drive pathogenesis and key components may be potential drug targets. For instance, a prioritized ER-positive breast cancer network captures a number of ribosomal proteins highlighting the importance of protein synthesis in disease aetiology. Indeed, recent observations show that an ERα inhibitor induces tumour regression via blocking protein synthesis in ER-positive breast cancer (35). Importantly, the network flow properties of network routers, key targets and high impact genes from an information flow perspective provide clues to prioritize drug targets. Furthermore, further inspection of network motifs and prioritized subnetwork of these key genes allow researchers to prioritize appropriate drug targets by avoiding parallel, compensatory information flow paths that can pass through two distinct key genes. As a rule of thumb to prioritize drug targets, top key genes (network routers, key targets and high impact genes) that show overall high positive scores in disease phenotypes can be therapeutically targeted to inhibit associated flows. In addition to overall score, the paths with high positive correlation coefficients and high information flows connecting these top key genes should be the main paths to be inhibited by drugs in order to revert disease phenotypes.

Taken together, the NetDecoder platform, specifically the ability to examine the biological impact of different phenotypes, has broad research implications. Although in this study we use DEGs as sources and transcriptional regulators as default sinks (targets), users may define their own sources and sinks depending on their research interests. For instance, a researcher can define highly mutated genes as sources and genes in executing cell cycle as sinks to study how mutated genes exert their functional impact on cell cycle. A researcher can also define known drug targets as sources and genes in executing apoptosis as sinks to study which drug target would most likely cause cell death in a given phenotype. The power of the NetDecoder platform is that it allows researchers to perform comparative analyses on any pair of biological contexts or phenotypes using either microarray data or RNA-seq data.

Lastly, it is important to note biological networks including PPI are highly dynamic and undergo continuous rewiring events (36). *In silico* approaches to predict context-specific network rewiring events are still far from mature (37). Therefore, the topology of the PPI network used in this study is rather ‘static’ and does not capture network rewiring events under different circumstances. Another issue is that a PPI network can be tissue-specific (38) and dependent on alternative splicing events (39) that are not considered in the current work. In the future, we will integrate information from other omics layers, especially epigenetics and proteomics, to enhance the capability of NetDecoder in discovering phenotype-specific subnetworks and the associated key genes to better understand phenotypic properties at multi-omics levels. Although the process poses challenges, the inferred context-specific networks and key genes discovered can serve as roadmaps for researchers to design a systems-based approach to manipulate biological phenotypes such as via edgetic perturbations (40,41) in the near future. Insights learned will also enable researchers to ‘engineer’ therapeutic strategies to reverse a disease phenotype via manipulating the information flows through key genes.

In summary, we present a novel computational network biology platform that has broad utility and biological impact. The NetDecoder platform allows researchers to explore context-dependent biological properties using both microarray and RNA-seq data and, in principle, supports single-cell RNA-seq data as well. The platform is flexible allowing researchers to define any set of genes to query their context-specific roles. We envision NetDecoder will play a major role in uncovering context-specific roles of oncogenes in pan-cancer studies, deciphering key genes that drive pathological progression in other disease classes, and prioritizing drug targets given specific biological contexts.

## AVAILABILITY OF SUPPORTING DATA

The datasets supporting the results of this article are available in the www.NetDecoder.org repository.

## Supplementary Material

Supplementary Data

SUPPLEMENTARY DATA
