# Supplementary material for: NetDecoder: a network biology platform that decodes context-specific biological networks and gene activities
Source: Nucleic Acids Res. 2016 Mar 14;44(10):e100. doi: 10.1093/nar/gkw166 (PMC4889937; doi:10.1093/nar/gkw166)
Supplement: Supplementary Data [file gkw166_Supplementary_Data.zip › nar-03685-met-z-2015-File007.pdf]

SUPPLEMENTARY INFORMATION

NetDecoder: A Network Biology Platform that Decodes Context-Specific Biological Networks and Gene Activities

Authors: Edroaldo Lummertz da Rocha[1,†], Choong Yong Ung[1,†], Cordelia D. McGehee[1], Cristina Correia[1], and Hu Li[1,*]

Affiliation: [1]Department of Molecular Pharmacology and Experimental Therapeutics, Center for Individualized Medicine, Mayo Clinic College of Medicine, Rochester, MN 55905, USA.

†These authors contributed equally to this work.

***Corresponding author:**

Hu Li, Ph.D.

Department of Molecular Pharmacology and Experimental Therapeutics
Mayo Clinic College of Medicine
Gonda Building, 19-408
200 First Street SW
Rochester, MN 55905
Office: 1-507-293-1182
Fax: 1- 507-284-4455
E-mail: li.hu@mayo.edu

SUPPORTING INFORMATION FILES

The following additional data are available with the online version of this paper. Supporting Information has all the supplementary figures Figure S1 to Figure S18 included in the analysis, and a supporting note to provide further discussion on context-specific functionalities of common biological processes mediated by key genes in respect to breast cancers, dyslipidemia, and Alzheimer's disease (AD).

SUPPLEMENTARY FIGURE LEGENDS

Figure S1. The information flow profiles of ER-negative breast cancer edge-specific subnetwork. The subnetwork was derived from identified edges as shown in Figure 2B. Edge thickness represents the amount of edge flow. Additionally, edges are colored according to the directionality of Pearson correlation coefficient (PCC) (red for positive correlation and blue for negative correlation). Source genes are indicated as diamonds, intermediary proteins as circles, and target genes (sinks) as squares and are colored according to node flow difference (see gradient bar on the right). The size of gene nodes is proportional to the total node flow.

Figure S2. ER-positive breast cancer-specific edge-centered subnetwork. (**A**) Heatmap of differentially expressed genes (DEGs) across control and ER-positive breast cancer. (**B**) Total edge flow profiles in control and ER-positive breast cancer for edges with higher flows in disease than in control subnetworks. (**C**) Jaccard index to evaluate the similarity between edge-centered control and disease subnetworks and Venn diagrams showing the overlap across genes, edges, and paths. Pathway enrichment analysis for: i) gene signatures (DEGs), ii) key edge-centered subnetworks in control, and iii) disease. Pathway enrichment analysis for genes that compose disease-specific edge-centered subnetworks captures signaling pathways that are relevant to disease etiology. No significant enrichment was detected for the gene signature. (**D**) Heatmap showing node flow differences across control and ER-positive breast cancer for top 20 network

routers and key target genes showing high node flow difference (red) and low node flow difference (blue).

Figure S3. Dyslipidemia heterozygote-specific edge-centered subnetwork. (**A**) Heatmap of differentially expressed genes (DEGs) across control and dyslipidemia heterozygote. (**B**) Total edge flow profiles in control and dyslipidemia heterozygote for edges with higher flows in disease than in control subnetworks. (**C**) Jaccard index to evaluate the similarity between edge-centered control and disease subnetworks and Venn diagrams showing the overlap across genes, edges, and paths. Pathway enrichment analysis for: i) gene signatures (DEGs) (red), ii) key edge-centered subnetworks in control (blue) and iii) disease (green). Pathway enrichment analysis for genes that compose disease-specific, edge-centered subnetworks captures signaling pathways that are relevant to disease etiology. No significant enrichment was detected for the gene signature. (**D**) Heatmap showing node flow differences across control and dyslipidemia heterozygote for top 20 network routers and key target genes showing high node flow difference (red) and low node flow difference (blue).

Figure S4. Dyslipidemia homozygote-specific edge-centered subnetwork. (**A**) Heatmap of differentially expressed genes (DEGs) across control and dyslipidemia homozygote. (**B**) Total edge flow profiles in control and dyslipidemia homozygote for edges with higher flows in disease than in control subnetworks. (**C**) Jaccard index to evaluate the similarity between edge-centered control and disease subnetworks and Venn diagrams showing the overlap across genes, edges, and paths. Pathway enrichment analysis for: i) gene signatures (DEGs) (red), ii) key edge-centered subnetworks in control (blue) and iii) disease (green). Pathway enrichment analysis for genes that compose disease-specific, edge-centered subnetworks captures signaling pathways that are relevant to disease etiology. No significant enrichment was detected for the gene

signature. (**D**) Heatmap showing node flow differences across control and dyslipidemia homozygote for top 20 network routers and key target genes showing high node flow difference (red) and low node flow difference (blue).

Figure S5. AD incipient-specific edge-centered subnetwork. (**A**) Heatmap of differentially expressed genes (DEGs) across control and AD incipient stage. (**B**) Total edge flow profiles in control and dyslipidemia homozygote for edges with higher flows in disease than in control subnetworks. (**C**) Jaccard index to evaluate the similarity between edge-centered control and disease subnetworks and Venn diagrams showing the overlap across genes, edges, and paths. Pathway enrichment analysis for: i) gene signatures (DEGs) (red), ii) key edge-centered subnetworks in control (blue) and iii) disease (green). Pathway enrichment analysis for genes that compose disease-specific, edge-centered subnetworks captures signaling pathways that are relevant to disease etiology. No significant enrichment was detected for the gene signature. (**D**) Heatmap showing node flow differences across control and AD incipient for top 20 network routers and key target genes showing high node flow difference (red) and low node flow difference (blue).

Figure S6. AD moderate-specific edge-centered subnetwork. (**A**) Heatmap of differentially expressed genes (DEGs) across control and AD moderate stage. (**B**) Total edge flow profiles in control and dyslipidemia homozygote for edges with higher flows in disease than in control subnetworks. (**C**) Jaccard index to evaluate the similarity between edge-centered control and disease subnetworks and Venn diagrams showing the overlap across genes, edges, and paths. Pathway enrichment analysis for: i) gene signatures (DEGs) (red), ii) key edge-centered subnetworks in control (blue) and iii) disease (green). Pathway enrichment analysis for genes that compose disease-specific, edge-centered subnetworks captures signaling pathways that are

relevant to disease etiology. No significant enrichment was detected for the gene signature. (**D**) Heatmap showing node flow differences across control and AD moderate for top 20 network routers and key target genes showing high node flow difference (red) and low node flow difference (blue).

Figure S7. AD severe-specific edge-specific subnetwork. (**A**) Heatmap of differentially expressed genes (DEGs) across control and AD severe stage. (**B**) Total edge flow profiles in control and dyslipidemia homozygote for edges with higher flows in disease than in control subnetworks. (**C**) Jaccard index to evaluate the similarity between edge-centered control and disease subnetworks and Venn diagrams showing the overlap across genes, edges, and paths. Pathway enrichment analysis for: i) gene signatures (DEGs) (red), ii) key edge-centered subnetworks in control (blue) and iii) disease (green). Pathway enrichment analysis for genes that compose disease-specific, edge-centered subnetworks captures signaling pathways that are relevant to disease etiology. No significant enrichment was detected for the gene signature. (**D**) Heatmap showing node flow differences across control and AD severe for top 20 network routers and key target genes showing high node flow difference (red) and low node flow difference (blue).

Figure S8. The MAPK1 motif. (**A**) MAPK1 as high impact gene in ER-negative breast cancer; (**B**) high impact gene in dyslipidemia homozygote; and (**C**) key target in AD severe.

Figure S9. The CSNK2A1 motif. (**A**) CSNK2A1 as high impact gene in ER-negative breast cancer; (**B**) key target in dyslipidemia homozygote; and (**C**) key target in AD incipient.

Figure S10. The RARA motif. (**A**) RARA as high impact gene in ER-negative breast cancer; (**B**) high impact gene in dyslipidemia homozygote; and (**C**) key target in dyslipidemia heterozygote.

Figure S11. The HDAC1 motif. (**A**) HDAC1 as high impact gene in ER-negative breast cancer; (**B**) key target in dyslipidemia heterozygote; and (**C**) high impact gene in AD severe.

Figure S12. The TERF2IP motif. (**A**) TERF2IP in AD incipient control. (**B**) TERF2IP as key target in AD moderate; (**C**) as key target in AD severe.

Figure S13. The CALR motif. (**A**) CALR as key target in dyslipidemia homozygote; (**B**) key target in AD moderate; and (**C**) key target in AD severe.

Figure S14. The EEF1A1 motif. (**A**) EEF1A1 as high impact gene in ER-negative breast cancer; (**B**) high impact gene in dyslipidemia homozygote; and (**C**) high impact gene in AD severe.

Figure S15. The CTNNB1 motif. (**A**) CTNNB1 as key target in AD incipient; (**B**) key target in AD moderate; and (**C**) high impact gene in AD severe.

Figure S16. Analyses of NetDecoder Robustness. Venn diagrams showing overlap of paths (**A**) and impact genes (**B**) identified by NetDecoder using size of the functional neighborhood, SFN=0.91, 0.93, 0.97 and 0.99 when compared to results obtained with SFN=0.95 (default). The number of predicted paths detected under a certain SFN is shown in the table at the bottom. (**C-D**) Heatmaps showing node flow differences across control and ER-negative breast cancer for top 20 key target genes (**C**) and network routers (**D**) for SFN=0.91, 0.93, 0.95 (default) 0.97 and 0.99. High node flow difference indicates in red and low node flow difference in blue. (**E**) Total edge flow profiles in control and ER-negative breast cancer for edges with higher flows in disease than in control-specific subnetworks using SFN=0.91, 0.93, 0.95 (default) 0.97 and 0.99 in control and ER-negative breast cancer.

Figure S17. Assigning flow paths based on shared biological processes. To decide which paths leads to maximum flow at the lowest cost, the process-guided flow algorithm takes into account the overlap between the functional neighborhoods of protein 1 and protein 2, and such overlap

becomes part of the protein 1 functional neighborhood (GO1, GO2 and GO7). Note that the functional neighborhood of a given protein also includes GO terms of the protein itself. If the functional neighborhood of protein 1 shares any biological process with the functional neighborhood of protein 2 (GO1, GO3, GO4 and GO7), protein 2 is included in the selected flow path. On the contrary, when protein 4 is considered as a candidate protein in a new path, there is no biological process being shared with the functional neighborhood of protein 1. Consequently, the flow is not directed through protein 4.

Figure S18. Context-dependence of key genes associated with FOXM1. **(A)** FOXM1 as a key gene (high impact gene and key target) in the ER-negative breast cancer context. **(B)** FOXM1 is not a key gene in the ER-positive breast cancer context. The identities of key genes cannot be directly located in a PPI network, but properties dictated by the way information flows are conveyed along the context-specific PPI network can be.

Supplementary Discussion

This Supplementary Discussion provides further discussion on context-specific functionalities of common biological processes mediated by key genes in respect to breast cancer, dyslipidemia, and Alzheimer's disease (AD). Many of these common biological processes are frequently enriched in transcriptome analyses but their context-dependent functionalities are seldom discussed. Understanding context-dependent activities of common biological processes can illuminate how the same enriched biological processes can show marked different properties in different biological contexts and what are the key genes that modulate information flows in resulting context-dependent behaviors.

Disease-specific canonical cellular processes

MAPK cascade is one of the major canonical signaling components that cross-talks with many other signaling pathways and its altered activities have been implicated in a number of human diseases. MAPK1, which is the key executor of the MAPK cascade, is a high impact gene in both ER-negative breast cancer and dyslipidemia homozygote (Figure 3) but a key target in AD severe (Figure S6D). Both EGFR and receptor tyrosine kinase (RYK) show uncoordinated function (negative co-expression coefficient) with MAPK1 (Figure S8A) probably due to reduced levels of internalized receptors that attenuate receptor kinase signaling in cancer cells. This is consistent with activation of the MAPK cascade in cancers with general functional coordination of BRAF and RAF1 with MAPK1 in ER-negative breast cancer (Figure S8A). In contrast, BRAF, GRB1 and SOS1 show coordinated function (positive co-expression coefficient) in the control case and RAF1 shows uncoordinated function with MAPK1 in dyslipidemia homozygote (Figure S8B), which suggests deregulated MAPK1 cascade activity in dyslipidemia homozygote. AD severe also shows altered MAPK cascade activity distinct from that observed in cancer and dyslipidemia. For instance, Polo-like kinase 3 (PLK3), which regulates cell cycle, shows uncoordinated function with MAPK1 in AD severe case (Figure S8C) where their pathological roles in AD remain to be elucidated.

Regulation of cell cycle

Cell cycle is a key process in cell growth, differentiation, and tissue repair in response to stress. Deregulated cell cycle processes can lead to uncontrolled cell proliferation in cancers or cell death via apoptosis. Casein kinase 2 (CSNK2A1), which regulates cell proliferation, is a high impact genes in ER negative breast cancer (Figure 3A), and a key target in dyslipidemia (Figure 3B) and incipient AD (Figure 3C). This gene shows positive correlation with positive regulators

of cell cycle such as Aurora kinase A (AURKA), CDC25B, CDK1, G2 checkpoint kinase WEE2, chromatin remodeler chromobox homolog 4 (CBX4) and topoisomerase 2A (TOP2A) in ER-negative breast cancer (Figure S9A). This in general suggests enhanced cell proliferative activity. However, in the control case the function of CSNK2A1 is coordinated with centrosomal protein CEP57 that is not captured in ER-negative breast cancer (Figure S9A), implicating deregulation of the chromosomal segregation process in cancer. In dyslipidemia homozygote, CSNK2A1 in general shows positive functional correlations with elongation factors in protein synthesis such as EIF2S2, EIF3J, EIF5B and heat shock protein HSP90B1 (Figure S9B). In incipient AD, CSNK2A1 shows functional coordination with heat shock protein HSP90AB1 and complement component C1R (Figure S9C). Our results suggest CSNK2A1 acts as cell cycle regulator in breast cancer, but its function is redirected to the modulation of protein fidelity in dyslipidemia and AD. However, the pathological roles of the functional coordination in the pathogenesis of dyslipidemia homozygote and AD remain unclear.

Regulation of transcriptional activities

Regulation of gene expression via transcriptional processes in response to environmental cues and cellular stress constantly takes place in all cell types. Altered interactions in transcription factors, co-activators, repressors, mediators, and RNA polymerase subunits are key events that lead to differential gene expression in cells of different biological contexts. Among transcription regulators, nuclear receptors are capable of regulating myriad cellular processes such as sensing cellular cues (in the form of steroid hormones), modulating homeostasis, and controlling metabolism. Retinoic acid receptor alpha (RARA) is a nuclear receptor that is a high impact gene in ER-negative breast cancer and dyslipidemia homozygote (Figure 3) and is a key target in

dyslipidemia heterozygote (Figure S3D). As shown in Figure S10 (bar charts), we found an increase in the overall edge flow of RARA in ER-negative breast cancer but reduced overall edge flows in both types of dyslipidemia. It is interesting to note that the transcription factor RUNX1, whose gene expression had been reported to correlate with poor prognosis in triple-negative breast cancer (1), shows functional coordination with RARA (Figure S10A). Methyl-CpG-binding protein 1 (MBD1) and Methyl-CpG-binding protein 3 (MBD3) also show positive correlation with RARA. How these protein interactions affect the reading of methyl code on CpG islands in cancer cells remains to be elucidated. In dyslipidemia homozygote, it seems there is increased functional coordination of transcriptional mediator MED25 with RARA due to low flow difference value of MED25 but positive correlative flow with RARA (Figure S10B). Interestingly, the autophagy receptor sequestosome 1 (SQSTM1), which regulates activity of the NFkB pathway shows marked coordinated function with RARA (Figure S10B), implying there is altered NFkB pathway activity in dyslipidemia homozygote patients. It is also interesting to note that there is a negative correlation between RXRA and RARA in dyslipidemia homozygote (Figure S10C). This suggests there is a reduced level of the RXRA-RARA heterodimer that associates with a multiprotein complex containing transcription corepressors that induce histone acetylation, chromatin condensation and transcriptional suppression. Decreased RXRA-RARA heterodimers in dyslipidemia homozygote patients might be involved in promoting expression of genes that promote dyslipidemia progression. In the dyslipidemia heterozygote case, the transcriptional mediator MED25 shows marked functional coordination with RARA than that of the homozygote case (Figure S10C). Unlike the homozygote case, in dyslipidemia heterozygote the methyl-CpG-binding protein 3 (MBD3) shows positive correlation with RARA that its role in disease etiology remained to be studied.

Chromatin remodeling, epigenetic and genome integrity

Epigenetic regulation can be mediated via methylating chromosomal DNA on the CpG islands or acetylating and methylating histones that wrap the chromatin. Epigenetic modifications can alter the structure of chromatin organization and change epigenetic states by remodeling chromatin structures leading to altered gene expression regulation. Key players in epigenetic regulation are DNA methyltransferases (DNMTs), histone acetyltransferases (HATs), and histone deacetylases (HDACs). Also, instability of a genome is the hallmark of many human common diseases. For instance, DNA damage and repair have been reported in atherosclerosis and metabolic syndromes (2, 3).

HDAC1 is a high impact gene in ER-negative breast cancer (Figure 3*A*). DNMT3A and DNMT3B, which are responsible for unmethylated CpG island methylation, and SMARCA4 and SMARCA1, which regulate chromatin remodeling, show negative correlations with HDAC1 in ER-negative breast cancer (Figure S11A). Given recent reports that inhibiting HDAC can sensitize cancer cells to radiotherapy (4), the fact that DNA topoisomerase II alpha (TOP2A) shows uncoordinated function with HDAC1 in ER-negative breast cancer (Figure S11A), might shed light on the mechanism behind this recent finding. For dyslipidemia heterozygote, RAP1A, a Ras oncogene family protein that regulates blood vessel formation, is found to show preferential functional coordination with HDAC1 (Figure S11B).

Histone acetylation catalyzed by histone acetyltransferases (HATs) on chromatin in the hippocampus corresponds with contextual learning (5) and reduced acetylation levels correspond with impaired memory (6). HDACs reverse the process of histone acetylation on chromatin and inhibiting their activities increases histone acetylation and enhances hippocampal-dependent

memory formation (7). Our finding that HDAC1 is a high impact gene in AD severe (Figure 3C) is consistent with the above observations. KDM5A, an enzyme that specifically demethylates lysine-4 of histone H3, shows positive correlation with HDAC1 in AD severe (Figure S11C). In contrast, BRCA1, a regulator of genome integrity, negatively correlates with HDAC1, which may lead to reduced DNA repair in AD severe patients. Interestingly, IKZF1, which regulates hematopoietic cell differentiation, shows uncoordinated function with HDAC1 (Figure S11C) that we suspect might cause deregulated blood supply in the brain of AD severe patients (8).

Telomeric repeat binding factor 2 (TERF2IP), which is involved in telomere length regulation, is a key target for AD moderate and severe states (Figure S6D, S7D). This highlights the importance of telomere maintenance in the pathogenesis of AD. On the other hand, our result suggests TERF2IP does not play a significant role in the development of incipient AD (Figure S12A) but begins to show its contribution as key targets in the moderate and severe stages of AD. General reduced overall edge flow and negative correlations of DNA damage response genes RAD50 and PML with TERF2IP in AD moderate and severe, respectively, (Figure S12B, S12C) suggest enhanced telomere damage in AD patients.

Unfolded protein responses

Correctly folded protein is essential for cellular function and accumulation of misfolded proteins inside a cell can cause cellular toxicity. In response to protein misfolding, a cell evokes unfolded protein responses (UPRs) to refold or degrade misfolded proteins. Molecular chaperones such as heat shock proteins and components of ubiquitin-proteasome system are keys to regulating the fidelity of the protein homeostasis inside a cell.

Calreticulin (CALR), a calcium-binding chaperone that promotes folding, is a key target for dyslipidemia homozygote (Figure S4D), AD moderate (Figure S6D), and AD severe (Figure S7D). This protein shows negative functional correlation with CUL7, a key protein that is required to regulate microtubule dynamics and genome integrity in dyslipidemia homozygote (Figure S13A), suggesting "disconnected" repairing processes mediated by CALR on microtubule and damaged DNA in the pathogenesis of dyslipidemia. HSP90B1 and GANAB, which regulate glycogen storage, show positive correlations with CALR in the dyslipidemia homozygote control case (Figure S13A) indicating folding and proper functioning of these proteins is important to rescue dyslipidemia. In the AD moderate and severe control cases, there are positive functional correlations between HSPB1 and ANP32B, which is an anti-apoptotic protein with CALR (Figure S13B, C), suggesting the involvement of apoptosis in AD pathogenesis. Interestingly, tumor necrosis factor receptor-associated factor-6 (TRAF6), which is an E3 ubiquitin ligase, is the top high impact gene for AD severe (Figure 3). TRAF6 had been shown to be essential for polyubiquitination of TrkA (a NGF receptor) when the complex TRAF6-p75 (NTR) is bound with TrkA-p62 complex upon NGF stimulation, leading to cell survival and neuronal differentiation. However, in AD the ubiquitination of TrkA is attenuated leading to apoptosis (9). In our study, we found uncoordinated function between TRAF6 with CALR in AD severe case (Figure S13C) suggesting increased misfolded states of TRAF6 in AD patients.

Regulation of protein synthesis

The rate of protein synthesis varies greatly among different tissues (10). Differences in the rate of protein synthesis are in fact intricate homeostatic regulations corresponding to diverse

functional states of a cell (11). Modulation of the assembly of ribosomal subunits contributes to differences in protein synthesis (12). Tightly regulated protein synthesis promotes tissue homeostasis by preventing uncontrolled cell proliferation. This is evidenced by the fact that cancer cells synthesize proteins more rapidly than normal cells (13) and suppressed protein synthesis could halt tumorigenesis (14).

The protein synthesis elongation factor EEF1A1 is a high impact gene in ER-negative breast cancer, dyslipidemia homozygote, and AD severe (Figure 3). There is preferential functional coordination between heat shock proteins (HSP90AA1) and ribosomal subunits in EEF1A1 in the control case of ER-negative breast cancer (Figure S14A), and this is also the general cases for dyslipidemia homozygote controls (Figure S14B) and AD severe control (Figure S14C) cases implying a more regulated protein synthesis, folding, and degradation in normal cases than in disease states. GAPDH (glyceraldehyde-3-phosphate dehydrogenase) and ENO3 (enolase 3), which are key metabolic regulators show positive and negative functional correlation with EEF1A1, respectively in dyslipidemia homozygote (Figure S14B). How these key metabolic regulators affect protein synthesis in driving pathogenesis of dyslipidemia remain to be studied. On the other hand, IMMT, which is an inner mitochondria membrane protein shows high values of flow difference in AD severe, suggesting malfunction of mitochondrial-mediated processes in AD pathogenesis. Interestingly, high values of node flow difference but negative functional correlation between IMMT and EEF1A1 shows their dissociated function in the AD severe case (Figure S14C). This observation might provide clues on how mitochondrial-driven bioenergetics processes can affect the progression of AD.

Cell-cell adhesion, integrity of cytoskeletal organization, and intracellular trafficking

Cell-cell adhesion provides the necessary avenue to support the cellular microenvironment. Deregulation of cytoskeletal organization is known to play a key role in many human diseases such as AD with the brain of patients marked by extensive extracellular β-amyloid (Aβ) plaques and intracellular neurofibrillary tangles (15, 16). We found amyloid-β precursor protein (APP) and heat shock proteins (HSP90AB1, HSP1A1, HSPA8) generally show functional coordination with catenin (CTNNB1) in AD control cases (Figure S15A). This suggests these functions are deregulated in AD. Interestingly, transcriptional regulator TCF4 plays an important role in nervous system development and also shows functional coordination with CTNNB1 in AD control cases (Figure S15C), indicating that there is a loss of neuronal function in AD patients' brains.

REFERENCES FOR SUPPLEMENTARY INFORMATION

1. Ferrari, N., Mohammed, Z.M., Nixon, C., Mason, S.M., Mallon, E., McMillan, D.C., Morris, J.S., Cameron, E.R., Edwards, J., Blyth, K. (2014) Expression of RUNX1 correlates with poor patient prognosis in triple negative breast cancer. *PLoS One*, **9**, e100759.
2. Cervelli, T., Borghini, A., Galli, A., Andreassi, M.G. (2012) DNA damage and repair in atherosclerosis: current insights and future perspectives. *Int. J. Mol. Sci.*, **13**, 16929-16944.
3. Mercer, J.R., Cheng, K.K., Figg, N., Gorenne, I., Mahmoudi, M., Griffin, J., Vidal-Puig, A., Logan, A., Murphy, M.P., Bennett, M. (2010) DNA damage links mitochondrial dysfunction to atherosclerosis and the metabolic syndrome. *Circ. Res.*, **107**, 1021-1031.
4. Lu, Y.S., Chou, C.H., Tzen, K.Y., Gao, M., Cheng, A.L., Kulp, S.K., Cheng, J.C. (2012) Radiosensitizing effect of a phenylbutyrate-derived histone deacetylase inhibitor in hepatocellular carcinoma. *Int. J. Radiat. Oncol. Biol. Phys.*, **83**, e181-9.
5. Levenson, J.M., O'Riordan, K.J., Brown, K.D., Trinh, M.A., Molfese, D.L., Sweatt, J.D. (2004) Regulation of histone acetylation during memory formation in the hippocampus. *J. Biol. Chem.*, **279**, 40545-40559.
6. Korzus, E., Rosenfeld, M.G., Mayford, M. (2004) CBP histone acetyltransferase activity is a critical component of memory consolidation. *Neuron*, **24**, 961-972.
7. Guan, J.S., Haggarty, S.J., Giacometti, E., Dannenberg, J.H., Joseph, N., Gao, J., Nieland, T.J., Zhou, Y., Wang, X., Mazitschek, R., Bradner, J.E., DePinho, R.A., Jaenisch, R.,Tsai, L.H. (2009) HDAC2 negatively regulates memory formation and synaptic plasticity. *Nature*, **459**, 55-60.

8. Iadecola, C. (2004) Neurovascular regulation in the normal brain and in Alzheimer's disease. *Nat. Rev. Neurosci.*, **5**, 347-360.
9. Zheng, C., Geetha, T., Gearing, M., Babu, JR. (2015) Amyloid β-abrogated TrkA ubiquitination in PC12 cells analogous to Alzheimer's disease. *J. Neurochem.*, **133**, 919-925.
10. Garlick, P.J., Wernerman, J., McNurlan, M.A., Heys, S.D. (1991) Organ-specific measurements of protein turnover in man. *Proc. Nutr. Soc.*, **50**, 217-225.
11. Buszczak, M., Signer, R.A., Morrison, S.J. (2014) Cellular differences in protein synthesis regulate tissue homeostasis. *Cell*, **159**, 242-251.
12. Kondrashov, N., Pusic, A., Stumpf, C.R., Shimizu, K., Hsieh, A.C., Xue, S., Ishijima, J., Shiroishi, T., Barna, M. (2011) Ribosome-mediated specificity in Hox mRNA translation and vertebrate tissue patterning. *Cell*, **145**, 383-397.
13. Silvera, D., Formenti, S.C., Schneider, R.J. (2010) Translational control in cancer. *Nat. Rev. Cancer*, **10**, 254-266.
14. Barna, M., Pusic, A., Zollo, O., Costa, M., Kondrashov, N., Rego, E., Rao, P.H., Ruggero, D. (2008) Suppression of Myc oncogenic activity by ribosomal protein haploinsufficiency. *Nature*, **456**, 971-975.
15. Dickson, D.W. (1997) Neuropathological diagnosis of Alzheimer's disease: a perspective from longitudinal clinicopathological studies. *Neurobiol. Aging*, **18**, S21-S26.
16. Braak, H., Braak, E. (1998) Evolution of neuronal changes in the course of Alzheimer's disease. *J. Neural. Transm. Suppl.*, **53**, 127-140.

Figure S1

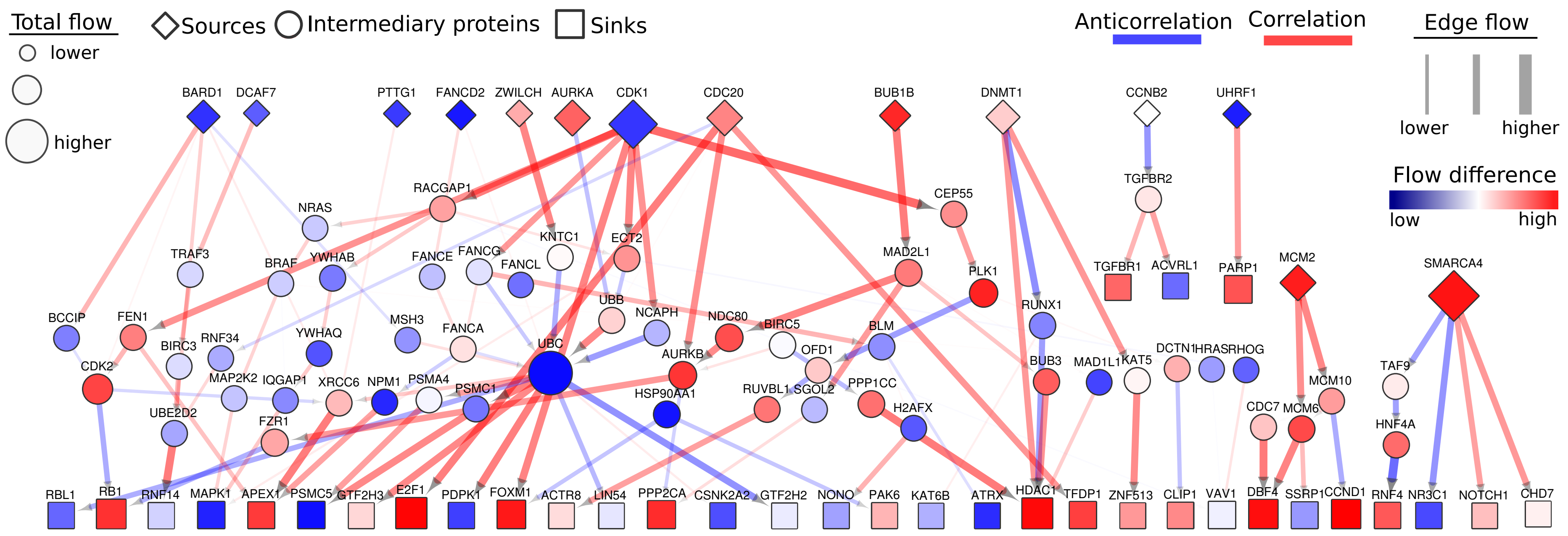
Total flow
lower
higher
Sources
Intermediary proteins
Sinks
Anticorrelation
Correlation
Edge flow
lower
higher
Flow difference
low
high
BARD1
DCAF7
PTTG1
FANCD2
ZWILCH
AURKA
CDK1
CDC20
BUB1B
DNMT1
CCNB2
UHRF1
TGFBR2
RACGAP1
CEP55
NRAS
KNTC1
ECT2
MAD2L1
TRAF3
BRAF
YWHAB
FANCE
FANCG
FANCL
PLK1
TGFBR1
ACVRL1
PARP1
MCM2
SMARCA4
BCCIP
FEN1
MSH3
FANCA
UBB
NCAPH
NDC80
BIRC5
BLM
RUNX1
BIRC3
RNF34
YWHAQ
UBC
OFD1
AURKB
BUB3
MAD1L1
KAT5
DCTN1
HRAS
RHOG
TAF9
CDK2
MAP2K2
IQGAP1
XRCC6
NPM1
PSMA4
PSMC1
HSP90AA1
RUVBL1
SGOL2
PPP1CC
MCM10
UBE2D2
FZR1
H2AFX
CDC7
MCM6
HNF4A
RBL1
RB1
RNF14
MAPK1
APEX1
PSMC5
GTF2H3
E2F1
PDPK1
FOXM1
ACTR8
LIN54
PPP2CA
CSNK2A2
GTF2H2
NONO
PAK6
KAT6B
ATRX
HDAC1
TFDP1
ZNF513
CLIP1
VAV1
DBF4
SSRP1
CCND1
RNF4
NR3C1
NOTCH1
CHD7

Figure S2

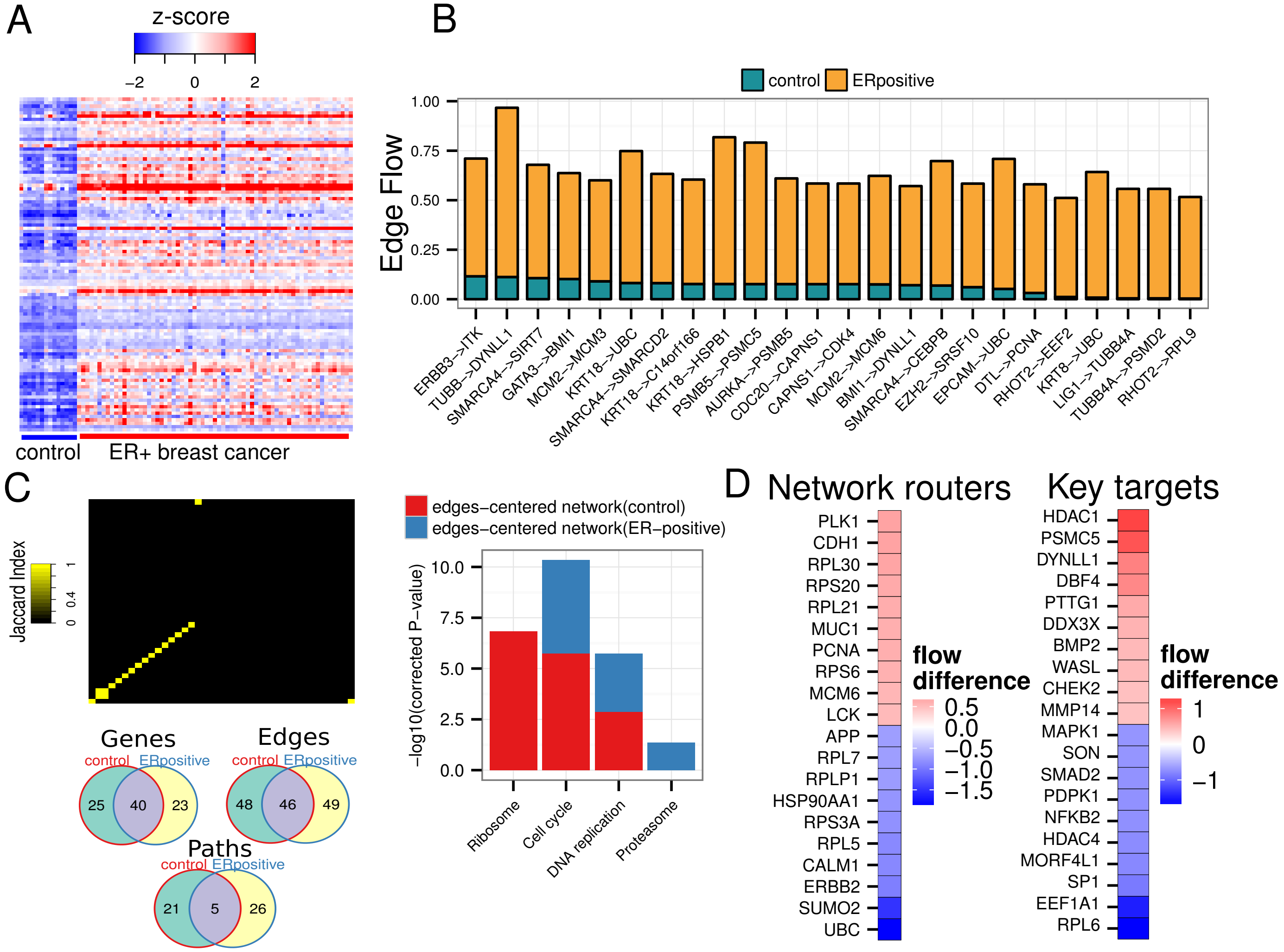
A
z-score
−2
0
2
control
ER+ breast cancer
B
control
ERpositive
Edge Flow
1.00
0.75
0.50
0.25
0.00
ERBB3−>ITK
TUBB−>DYNLL1
SMARCA4−>SIRT7
GATA3−>BMI1
MCM2−>MCM3
KRT18−>UBC
SMARCA4−>SMARCD2
KRT18−>C14orf166
KRT18−>HSPB1
PSMB5−>PSMC5
AURKA−>PSMB5
CDC20−>CAPNS1
CAPNS1−>CDK4
MCM2−>MCM6
BMI1−>DYNLL1
SMARCA4−>CEBPB
EZH2−>SRSF10
EPCAM−>UBC
DTL−>PCNA
RHOT2−>EEF2
KRT8−>UBC
LIG1−>TUBB4A
TUBB4A−>PSMD2
RHOT2−>RPL9
C
Jaccard Index
0
0.4
1
Genes
control
ERpositive
25
40
23
Edges
control
ERpositive
48
46
49
Paths
control
ERpositive
21
5
26
edges−centered network(control)
edges−centered network(ER−positive)
−log10(corrected P−value)
10.0
7.5
5.0
2.5
0.0
Ribosome
Cell cycle
DNA replication
Proteasome
D
Network routers
PLK1
CDH1
RPL30
RPS20
RPL21
MUC1
PCNA
RPS6
MCM6
LCK
APP
RPL7
RPLP1
HSP90AA1
RPS3A
RPL5
CALM1
ERBB2
SUMO2
UBC
flow difference
0.5
0.0
−0.5
−1.0
−1.5
Key targets
HDAC1
PSMC5
DYNLL1
DBF4
PTTG1
DDX3X
BMP2
WASL
CHEK2
MMP14
MAPK1
SON
SMAD2
PDPK1
NFKB2
HDAC4
MORF4L1
SP1
EEF1A1
RPL6
flow difference
1
0
−1

Figure S3

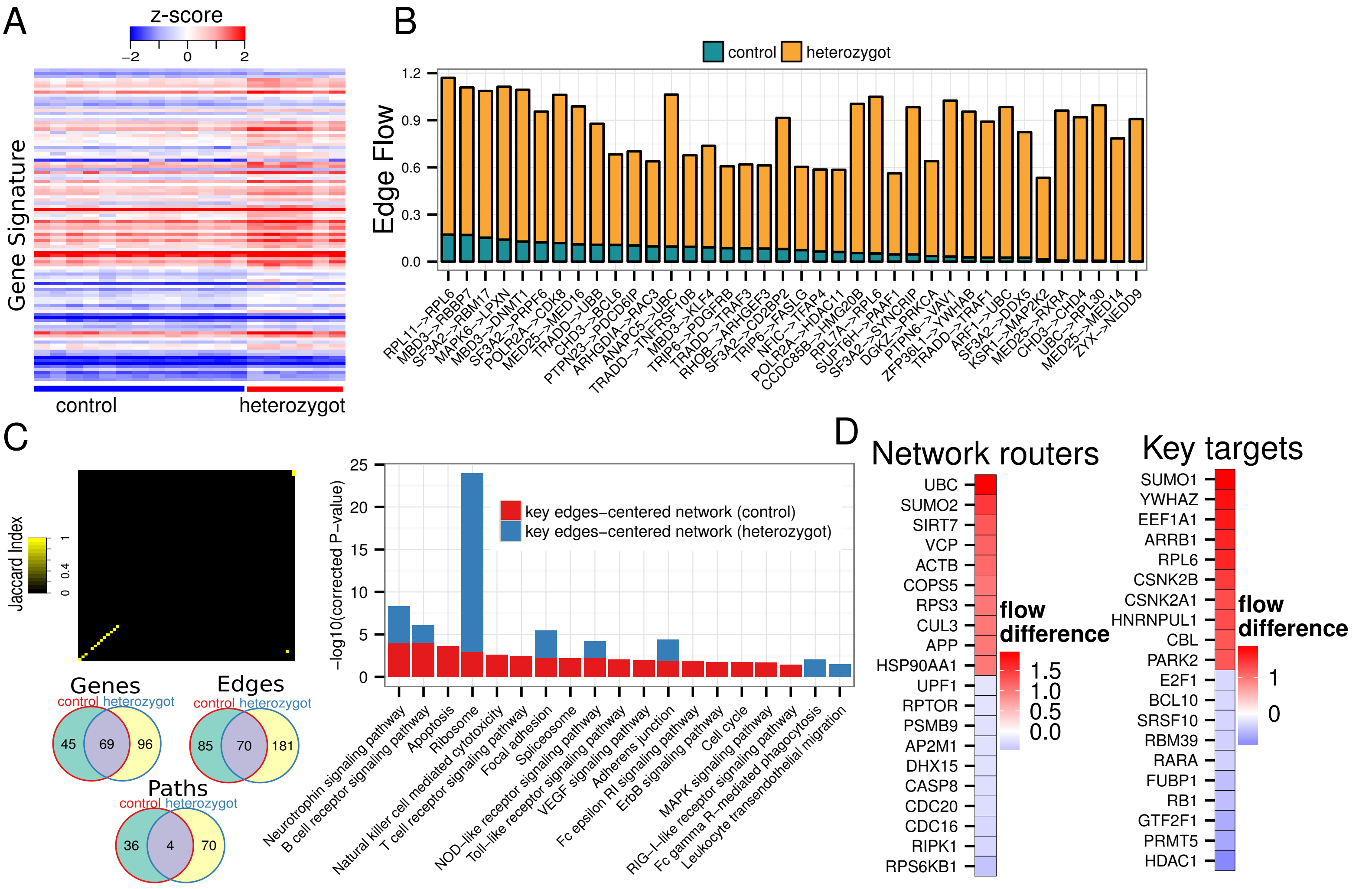

A
z-score
−2
0
2
Gene Signature
control
heterozygot
B
control
heterozygot
Edge Flow
1.2
0.9
0.6
0.3
0.0
RPL11−>RPL6
MBD3−>RBBP7
SF3A2−>RBM17
MAPK6−>LPXN
MBD3−>DNMT1
SF3A2−>PRPF6
POLR2A−>CDK8
MED25−>MED16
TRADD−>UBB
CHD3−>BCL6
PTPN23−>PDCD6IP
ARHGDIA−>RAC3
ANAPC5−>UBC
TRADD−>TNFRSF10B
MBD3−>KLF4
TRIP6−>PDGFRB
TRADD−>TRAF3
RHOB−>ARHGEF3
SF3A2−>CD2BP2
TRIP6−>FASLG
NFIC−>TFAP4
POLR2A−>HDAC11
CCDC85B−>HMG20B
RPL7A−>RPL6
SUPT6H−>PAAF1
SF3A2−>SYNCRIP
DGKZ−>PRKCA
PTPN6−>VAV1
ZFP36L1−>YWHAB
TRADD−>TRAF1
ARF1−>UBC
SF3A2−>DDX5
KSR1−>MAP2K2
MED25−>RXRA
CHD3−>CHD4
UBC−>RPL30
MED25−>MED14
ZYX−>NEDD9
C
Jaccard Index
1
0.4
0
Genes
control heterozygot
45
69
96
Edges
control heterozygot
85
70
181
Paths
control heterozygot
36
4
70
−log10(corrected P−value)
25
20
15
10
5
0
key edges−centered network (control)
key edges−centered network (heterozygot)
Neurotrophin signaling pathway
B cell receptor signaling pathway
Apoptosis
Ribosome
Natural killer cell mediated cytotoxicity
T cell receptor signaling pathway
Focal adhesion
Spliceosome
NOD−like receptor signaling pathway
Toll−like receptor signaling pathway
VEGF signaling pathway
Adherens junction
Fc epsilon RI signaling pathway
ErbB signaling pathway
Cell cycle
MAPK signaling pathway
RIG−I−like receptor signaling pathway
Fc gamma R−mediated phagocytosis
Leukocyte transendothelial migration
D
Network routers
UBC
SUMO2
SIRT7
VCP
ACTB
COPS5
RPS3
CUL3
APP
HSP90AA1
UPF1
RPTOR
PSMB9
AP2M1
DHX15
CASP8
CDC20
CDC16
RIPK1
RPS6KB1
flow difference
1.5
1.0
0.5
0.0
Key targets
SUMO1
YWHAZ
EEF1A1
ARRB1
RPL6
CSNK2B
CSNK2A1
HNRNPUL1
CBL
PARK2
E2F1
BCL10
SRSF10
RBM39
RARA
FUBP1
RB1
GTF2F1
PRMT5
HDAC1
flow difference
1
0

Figure S4

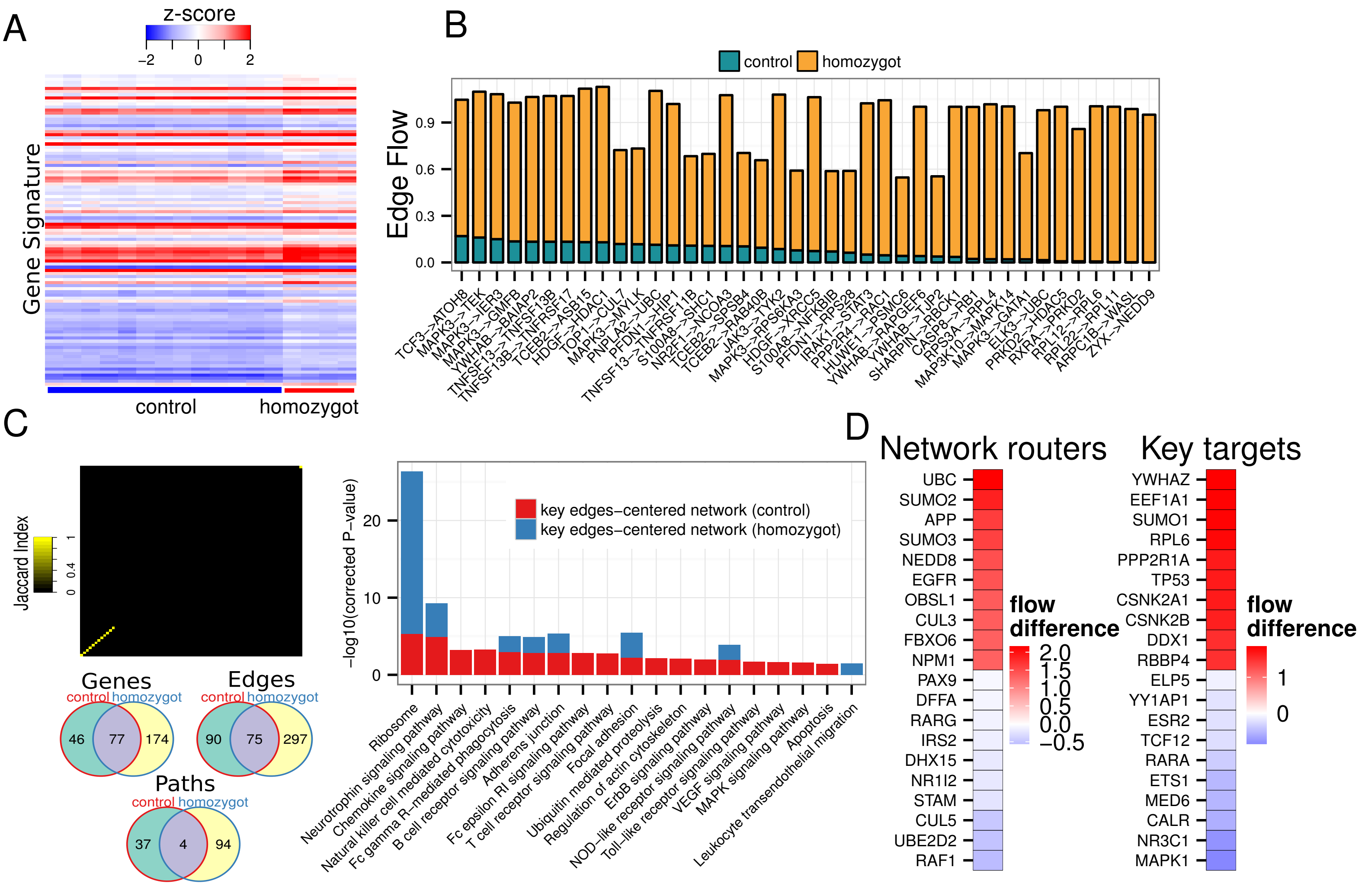

A
z-score
−2
0
2
Gene Signature
control
homozygot
B
control
homozygot
Edge Flow
0.0
0.3
0.6
0.9
TCF3−>ATOH8
MAPK3−>TEK
MAPK3−>IER3
MAPK3−>GMFB
YWHAB−>BAIAP2
TNFSF13−>TNFSF13B
TNFSF13B−>TNFRSF17
TCEB2−>ASB15
HDGF−>HDAC1
TOP1−>CUL7
MAPK3−>MYLK
PNPLA2−>UBC
PFDN1−>HIP1
TNFSF13−>TNFRSF11B
S100A8−>SHC1
NR2F1−>NCOA3
TCEB2−>SPSB4
TCEB2−>RAB40B
JAK3−>TYK2
MAPK3−>RPS6KA3
HDGF−>XRCC5
S100A8−>NFKBIB
PFDN1−>RPS28
IRAK1−>STAT3
PPP2R4−>RAC1
HUWE1−>PSMC6
YWHAB−>RAPGEF6
YWHAB−>TJP2
SHARPIN−>RBCK1
CASP8−>RB1
RPS3A−>RPL4
MAP3K10−>MAPK14
MAPK3−>GATA1
ELK3−>UBC
PRKD2−>HDAC5
RXRA−>PRKD2
RPL12−>RPL6
RPL22−>RPL11
ARPC1B−>WASL
ZYX−>NEDD9
C
Jaccard Index
0
0.4
1
Genes
control homozygot
46
77
174
Edges
control homozygot
90
75
297
Paths
control homozygot
37
4
94
−log10(corrected P−value)
0
10
20
key edges−centered network (control)
key edges−centered network (homozygot)
Ribosome
Neurotrophin signaling pathway
Chemokine signaling pathway
Natural killer cell mediated cytotoxicity
Fc gamma R−mediated phagocytosis
B cell receptor signaling pathway
Adherens junction
Fc epsilon RI signaling pathway
T cell receptor signaling pathway
Focal adhesion
Ubiquitin mediated proteolysis
Regulation of actin cytoskeleton
ErbB signaling pathway
NOD−like receptor signaling pathway
Toll−like receptor signaling pathway
VEGF signaling pathway
MAPK signaling pathway
Apoptosis
Leukocyte transendothelial migration
D
Network routers
UBC
SUMO2
APP
SUMO3
NEDD8
EGFR
OBSL1
CUL3
FBXO6
NPM1
PAX9
DFFA
RARG
IRS2
DHX15
NR1I2
STAM
CUL5
UBE2D2
RAF1
flow difference
2.0
1.5
1.0
0.5
0.0
−0.5
Key targets
YWHAZ
EEF1A1
SUMO1
RPL6
PPP2R1A
TP53
CSNK2A1
CSNK2B
DDX1
RBBP4
ELP5
YY1AP1
ESR2
TCF12
RARA
ETS1
MED6
CALR
NR3C1
MAPK1
flow difference
1
0

Figure S5

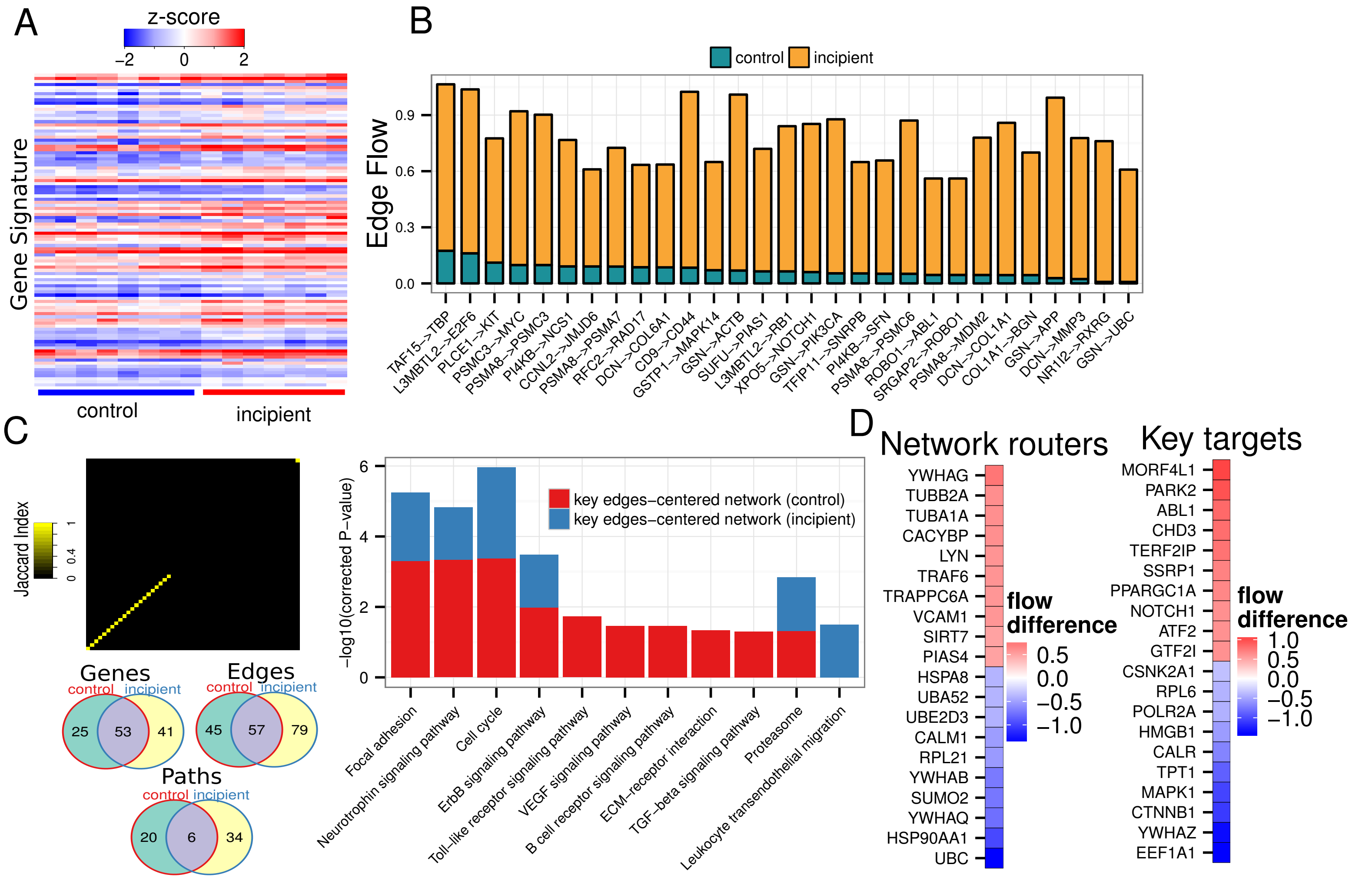
A
z-score
−2
0
2
Gene Signature
control
incipient
B
control
incipient
Edge Flow
0.0
0.3
0.6
0.9
TAF15−>TBP
L3MBTL2−>E2F6
PLCE1−>KIT
PSMC3−>MYC
PSMA8−>PSMC3
PI4KB−>NCS1
CCNL2−>JMJD6
PSMA8−>PSMA7
RFC2−>RAD17
DCN−>COL6A1
CD9−>CD44
GSTP1−>MAPK14
GSN−>ACTB
SUFU−>PIAS1
L3MBTL2−>RB1
XPO5−>NOTCH1
GSN−>PIK3CA
TFIP11−>SNRPB
PI4KB−>SFN
PSMA8−>PSMC6
ROBO1−>ABL1
SRGAP2−>ROBO1
PSMA8−>MDM2
DCN−>COL1A1
COL1A1−>BGN
GSN−>APP
DCN−>MMP3
NR1I2−>RXRG
GSN−>UBC
C
Jaccard Index
0
0.4
1
Genes
control
incipient
25
53
41
Edges
control
incipient
45
57
79
Paths
control
incipient
20
6
34
−log10(corrected P−value)
0
2
4
6
key edges−centered network (control)
key edges−centered network (incipient)
Focal adhesion
Neurotrophin signaling pathway
Cell cycle
ErbB signaling pathway
Toll−like receptor signaling pathway
VEGF signaling pathway
B cell receptor signaling pathway
ECM−receptor interaction
TGF−beta signaling pathway
Proteasome
Leukocyte transendothelial migration
D
Network routers
YWHAG
TUBB2A
TUBA1A
CACYBP
LYN
TRAF6
TRAPPC6A
VCAM1
SIRT7
PIAS4
HSPA8
UBA52
UBE2D3
CALM1
RPL21
YWHAB
SUMO2
YWHAQ
HSP90AA1
UBC
flow difference
0.5
0.0
−0.5
−1.0
Key targets
MORF4L1
PARK2
ABL1
CHD3
TERF2IP
SSRP1
PPARGC1A
NOTCH1
ATF2
GTF2I
CSNK2A1
RPL6
POLR2A
HMGB1
CALR
TPT1
MAPK1
CTNNB1
YWHAZ
EEF1A1
flow difference
1.0
0.5
0.0
−0.5
−1.0

Figure S6

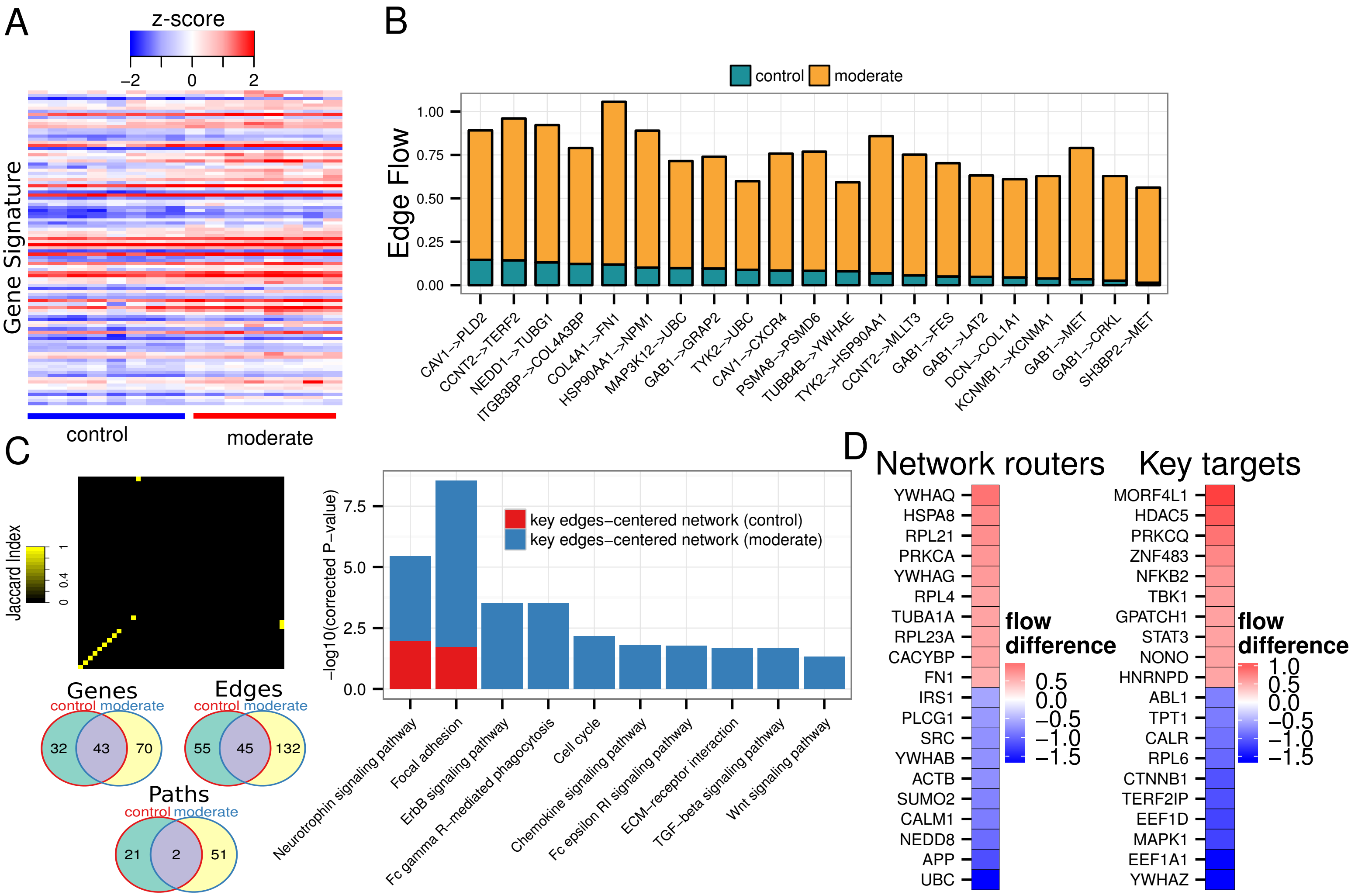

A
z-score
−2
0
2
Gene Signature
control
moderate
B
control
moderate
Edge Flow
1.00
0.75
0.50
0.25
0.00
CAV1->PLD2
CCNT2->TERF2
NEDD1->TUBG1
ITGB3BP->COL4A3BP
COL4A1->FN1
HSP90AA1->NPM1
MAP3K12->UBC
GAB1->GRAP2
TYK2->UBC
CAV1->CXCR4
PSMA8->PSMD6
TUBB4B->YWHAE
TYK2->HSP90AA1
CCNT2->MLLT3
GAB1->FES
GAB1->LAT2
DCN->COL1A1
KCNMB1->KCNMA1
GAB1->MET
GAB1->CRKL
SH3BP2->MET
C
Jaccard Index
0
0.4
1
Genes
control moderate
32
43
70
Edges
control moderate
55
45
132
Paths
control moderate
21
2
51
−log10(corrected P−value)
7.5
5.0
2.5
0.0
key edges−centered network (control)
key edges−centered network (moderate)
Neurotrophin signaling pathway
Focal adhesion
ErbB signaling pathway
Fc gamma R−mediated phagocytosis
Cell cycle
Chemokine signaling pathway
Fc epsilon RI signaling pathway
ECM−receptor interaction
TGF−beta signaling pathway
Wnt signaling pathway
D
Network routers
YWHAQ
HSPA8
RPL21
PRKCA
YWHAG
RPL4
TUBA1A
RPL23A
CACYBP
FN1
IRS1
PLCG1
SRC
YWHAB
ACTB
SUMO2
CALM1
NEDD8
APP
UBC
flow difference
0.5
0.0
−0.5
−1.0
−1.5
Key targets
MORF4L1
HDAC5
PRKCQ
ZNF483
NFKB2
TBK1
GPATCH1
STAT3
NONO
HNRNPD
ABL1
TPT1
CALR
RPL6
CTNNB1
TERF2IP
EEF1D
MAPK1
EEF1A1
YWHAZ
flow difference
1.0
0.5
0.0
−0.5
−1.0
−1.5

Figure S7

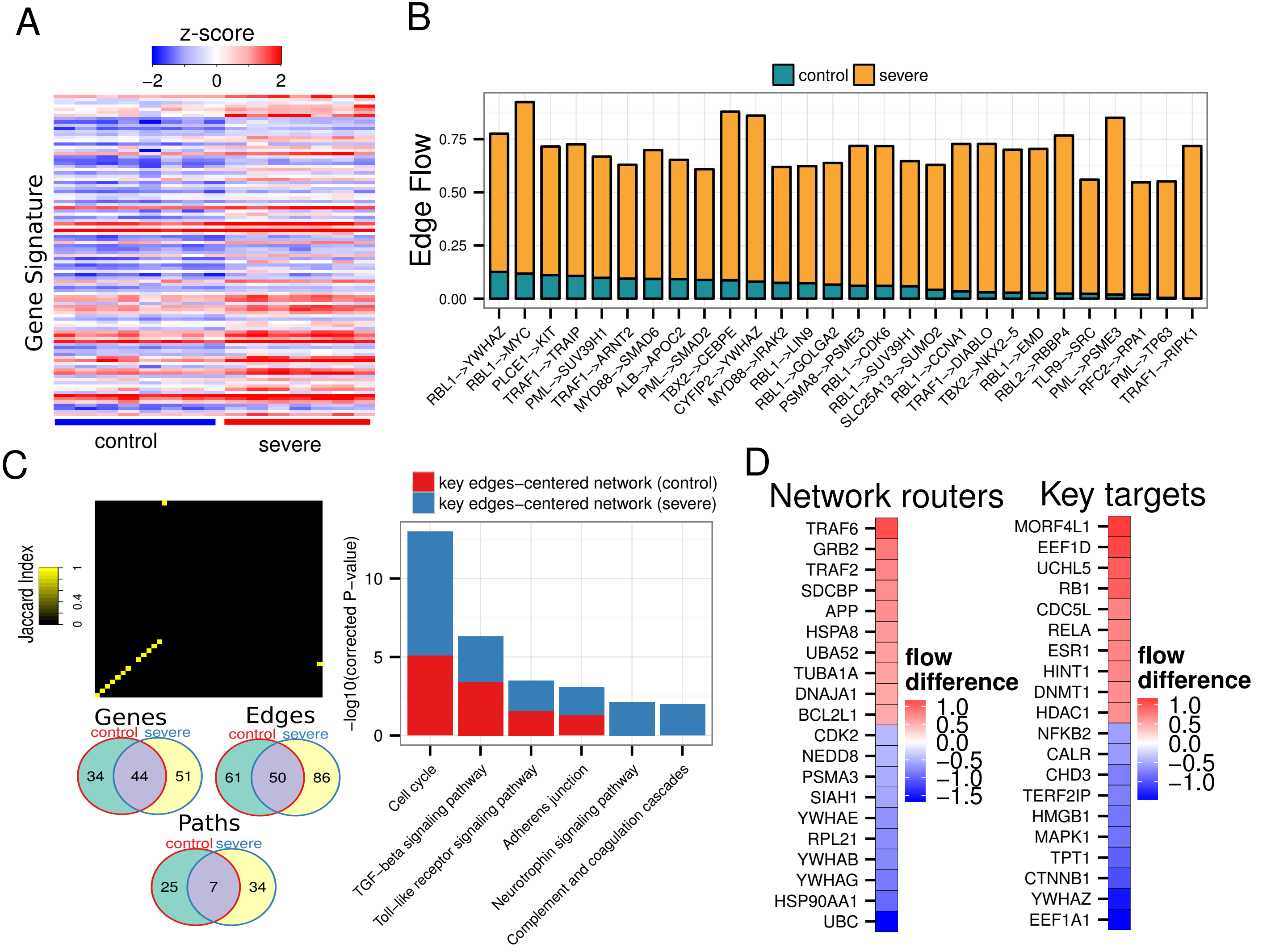

A
z-score
−2
0
2
Gene Signature
control
severe
B
control
severe
Edge Flow
0.00
0.25
0.50
0.75
RBL1−>YWHAZ
RBL1−>MYC
PLCE1−>KIT
TRAF1−>TRAIP
PML−>SUV39H1
TRAF1−>ARNT2
MYD88−>SMAD6
ALB−>APOC2
PML−>SMAD2
TBX2−>CEBPE
CYFIP2−>YWHAZ
MYD88−>IRAK2
RBL1−>LIN9
RBL1−>GOLGA2
PSMA8−>PSME3
RBL1−>CDK6
RBL1−>SUV39H1
SLC25A13−>SUMO2
RBL1−>CCNA1
TRAF1−>DIABLO
TBX2−>NKX2-5
RBL1−>EMD
RBL2−>RBBP4
TLR9−>SRC
PML−>PSME3
RFC2−>RPA1
PML−>TP63
TRAF1−>RIPK1
C
Jaccard Index
0
0.4
1
Genes
control
severe
34
44
51
Edges
control
severe
61
50
86
Paths
control
severe
25
7
34
key edges−centered network (control)
key edges−centered network (severe)
−log10(corrected P−value)
0
5
10
Cell cycle
TGF−beta signaling pathway
Toll−like receptor signaling pathway
Adherens junction
Neurotrophin signaling pathway
Complement and coagulation cascades
D
Network routers
TRAF6
GRB2
TRAF2
SDCBP
APP
HSPA8
UBA52
TUBA1A
DNAJA1
BCL2L1
CDK2
NEDD8
PSMA3
SIAH1
YWHAE
RPL21
YWHAB
YWHAG
HSP90AA1
UBC
flow difference
1.0
0.5
0.0
−0.5
−1.0
−1.5
Key targets
MORF4L1
EEF1D
UCHL5
RB1
CDC5L
RELA
ESR1
HINT1
DNMT1
HDAC1
NFKB2
CALR
CHD3
TERF2IP
HMGB1
MAPK1
TPT1
CTNNB1
YWHAZ
EEF1A1
flow difference
1.0
0.5
0.0
−0.5
−1.0

Figure S8

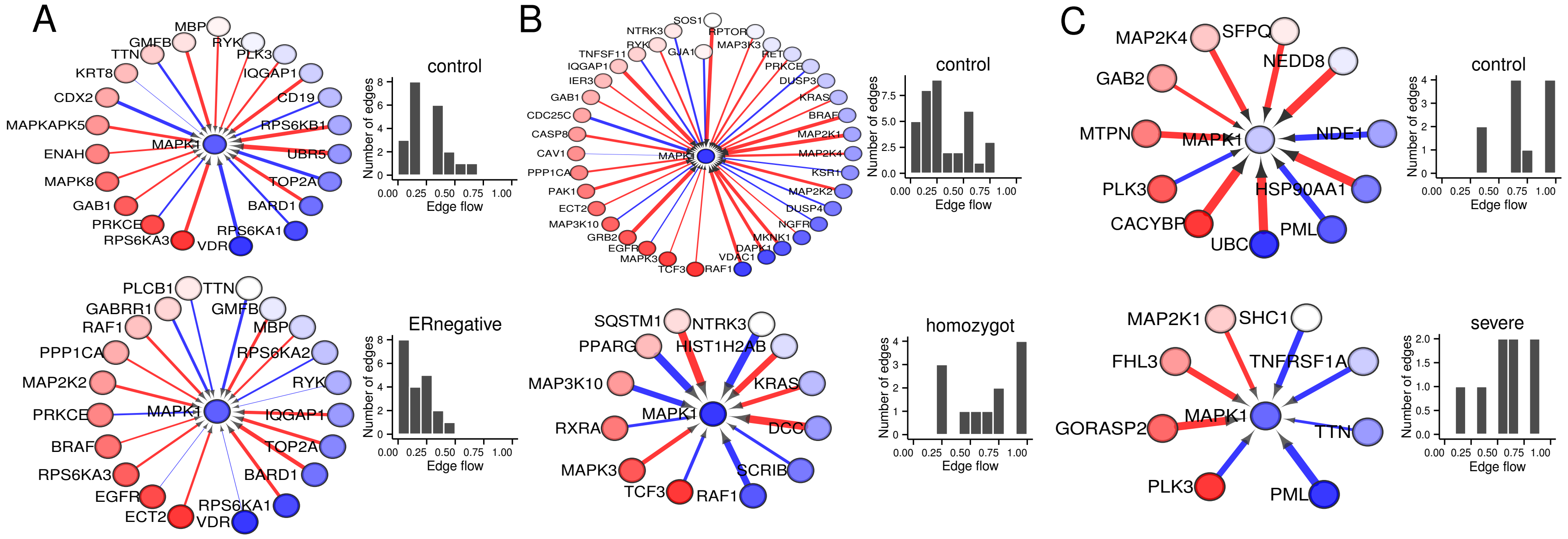
A
MBP
GMFB
RYK
TTN
PLK3
KRT8
IQGAP1
CDX2
CD19
MAPKAPK5
RPS6KB1
MAPK1
ENAH
UBR5
MAPK8
TOP2A
GAB1
BARD1
PRKCE
RPS6KA1
RPS6KA3
VDR
control
Number of edges
Edge flow
ERnegative
PLCB1
TTN
GABRR1
GMFB
RAF1
MBP
PPP1CA
RPS6KA2
MAP2K2
RYK
PRKCE
MAPK1
IQGAP1
BRAF
TOP2A
RPS6KA3
BARD1
EGFR
RPS6KA1
ECT2
VDR
B
SOS1
NTRK3
RPTOR
RYK
MAP3K3
TNFSF11
GJA1
RET
IQGAP1
PRKCE
IER3
DUSP3
GAB1
KRAS
CDC25C
BRAF
CASP8
MAP2K1
CAV1
MAP2K4
MAPK1
PPP1CA
KSR1
PAK1
MAP2K2
ECT2
DUSP4
MAP3K10
NGFR
GRB2
MKNK1
EGFR
DAPK1
MAPK3
VDAC1
TCF3
RAF1
SQSTM1
NTRK3
PPARG
HIST1H2AB
MAP3K10
KRAS
MAPK1
RXRA
DCC
MAPK3
SCRIB
TCF3
RAF1
homozygot
C
MAP2K4
SFPQ
NEDD8
GAB2
MTPN
MAPK1
NDE1
PLK3
HSP90AA1
CACYBP
PML
UBC
MAP2K1
SHC1
FHL3
TNFRSF1A
MAPK1
GORASP2
TTN
PLK3
PML
severe

Figure S9

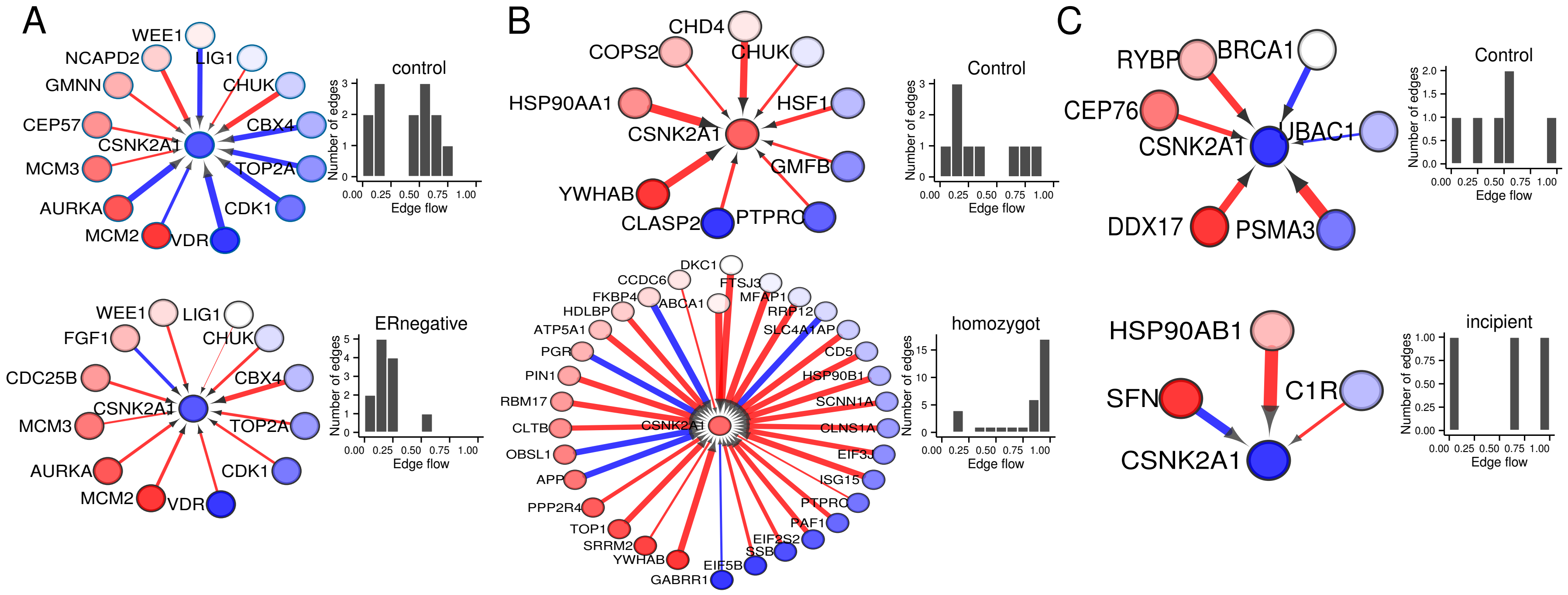

A
WEE1
NCAPD2
LIG1
GMNN
CHUK
CEP57
CBX4
CSNK2A1
MCM3
TOP2A
AURKA
CDK1
MCM2
VDR
control
Number of edges
Edge flow
WEE1
LIG1
FGF1
CHUK
CDC25B
CBX4
CSNK2A1
MCM3
TOP2A
AURKA
CDK1
MCM2
VDR
ERnegative
B
CHD4
COPS2
CHUK
HSP90AA1
HSF1
CSNK2A1
GMFB
YWHAB
CLASP2
PTPRC
Control
DKC1
CCDC6
FTSJ3
FKBP4
ABCA1
MFAP1
HDLBP
RRP12
ATP5A1
SLC4A1AP
PGR
CD5
PIN1
HSP90B1
RBM17
SCNN1A
CLTB
CSNK2A1
CLNS1A
OBSL1
EIF3J
APP
ISG15
PPP2R4
PTPRC
TOP1
PAF1
SRRM2
EIF2S2
YWHAB
SSB
EIF5B
GABRR1
homozygot
C
RYBP
BRCA1
CEP76
UBAC1
CSNK2A1
DDX17
PSMA3
Control
HSP90AB1
SFN
C1R
CSNK2A1
incipient

Figure S10

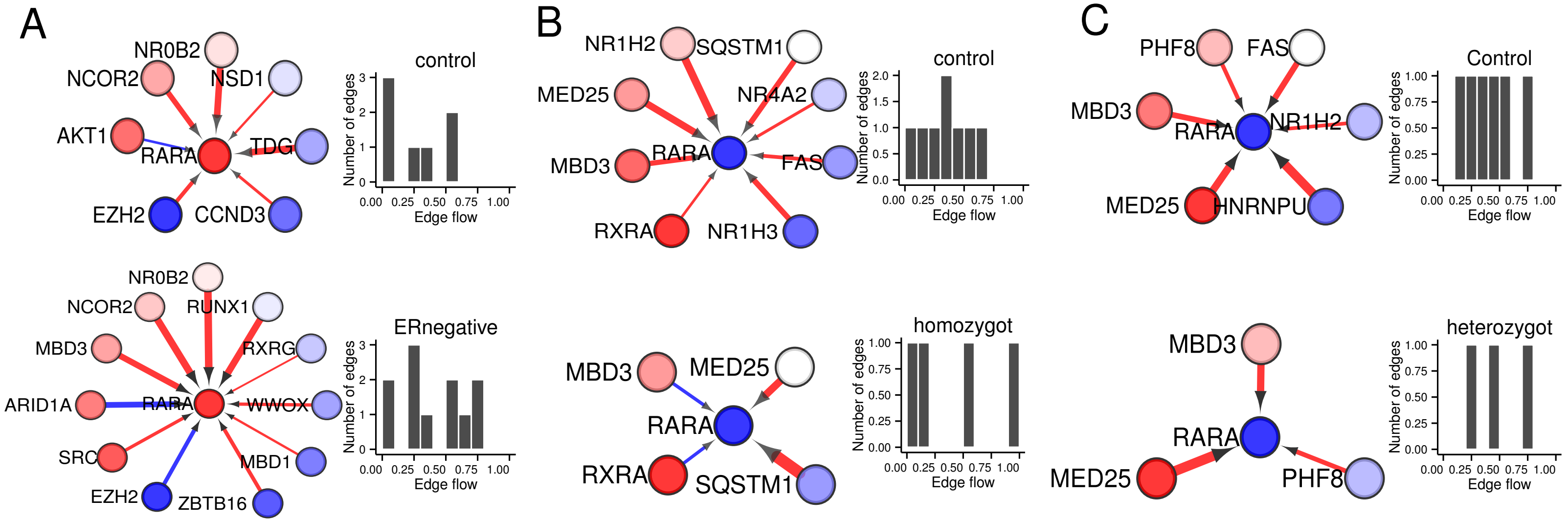
A
NR0B2
NCOR2
NSD1
AKT1
RARA
TDG
EZH2
CCND3
control
Number of edges
Edge flow
NR0B2
NCOR2
RUNX1
MBD3
RXRG
ARID1A
RARA
WWOX
SRC
MBD1
EZH2
ZBTB16
ERnegative
B
NR1H2
SQSTM1
MED25
NR4A2
RARA
MBD3
FAS
RXRA
NR1H3
control
MBD3
MED25
RARA
RXRA
SQSTM1
homozygot
C
PHF8
FAS
MBD3
RARA
NR1H2
MED25
HNRNPU
Control
MBD3
RARA
MED25
PHF8
heterozygot

Figure S11

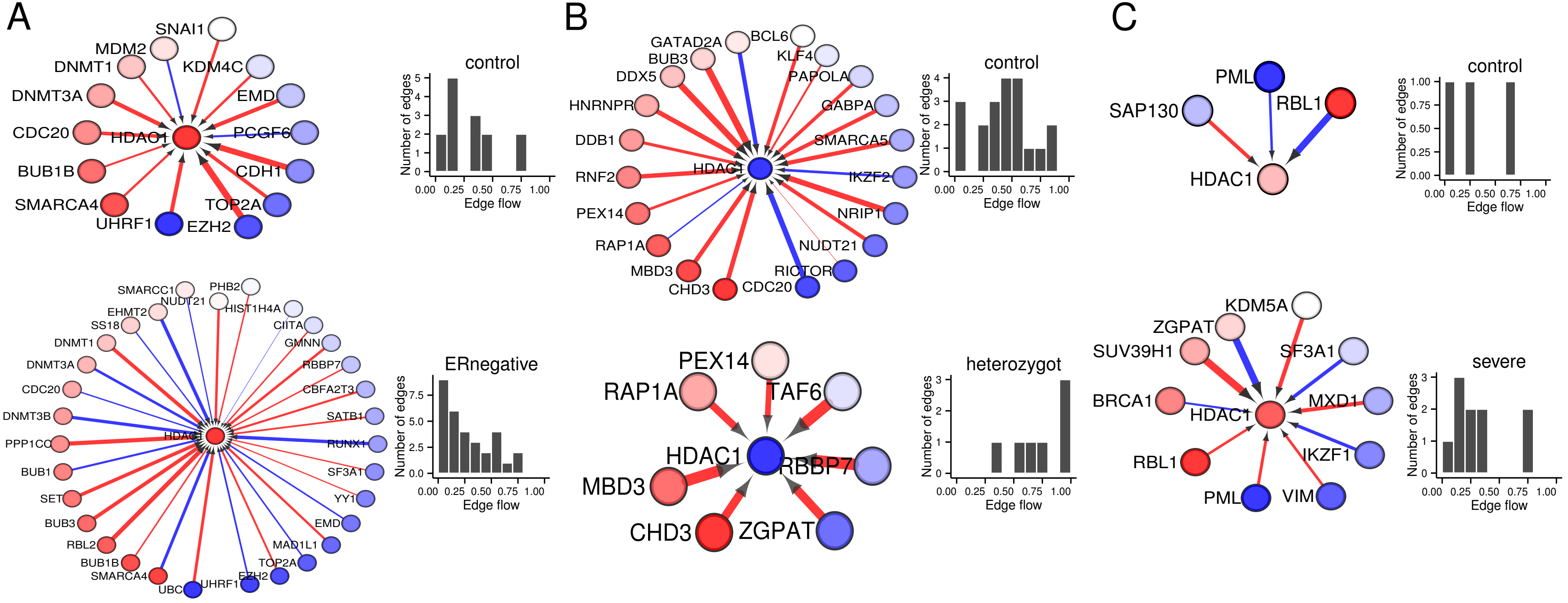

A
SNAI1
MDM2
DNMT1
KDM4C
DNMT3A
EMD
CDC20
HDAC1
PCGF6
BUB1B
CDH1
SMARCA4
TOP2A
UHRF1
EZH2
control
Number of edges
Edge flow
SMARCC1
PHB2
NUDT21
EHMT2
HIST1H4A
SS18
CIITA
DNMT1
GMNN
DNMT3A
RBBP7
CDC20
CBFA2T3
DNMT3B
SATB1
PPP1CC
RUNX1
BUB1
SF3A1
SET
YY1
BUB3
EMD
RBL2
MAD1L1
BUB1B
TOP2A
SMARCA4
EZH2
UBC
UHRF1
ERnegative
B
GATAD2A
BCL6
BUB3
KLF4
DDX5
PAPOLA
HNRNPR
GABPA
DDB1
SMARCA5
RNF2
HDAC1
IKZF2
PEX14
NRIP1
RAP1A
NUDT21
MBD3
RICTOR
CHD3
CDC20
control
PEX14
RAP1A
TAF6
HDAC1
RBBP7
MBD3
CHD3
ZGPAT
heterozygot
C
PML
SAP130
RBL1
HDAC1
control
KDM5A
ZGPAT
SUV39H1
SF3A1
BRCA1
MXD1
HDAC1
RBL1
IKZF1
PML
VIM
severe

Figure S12

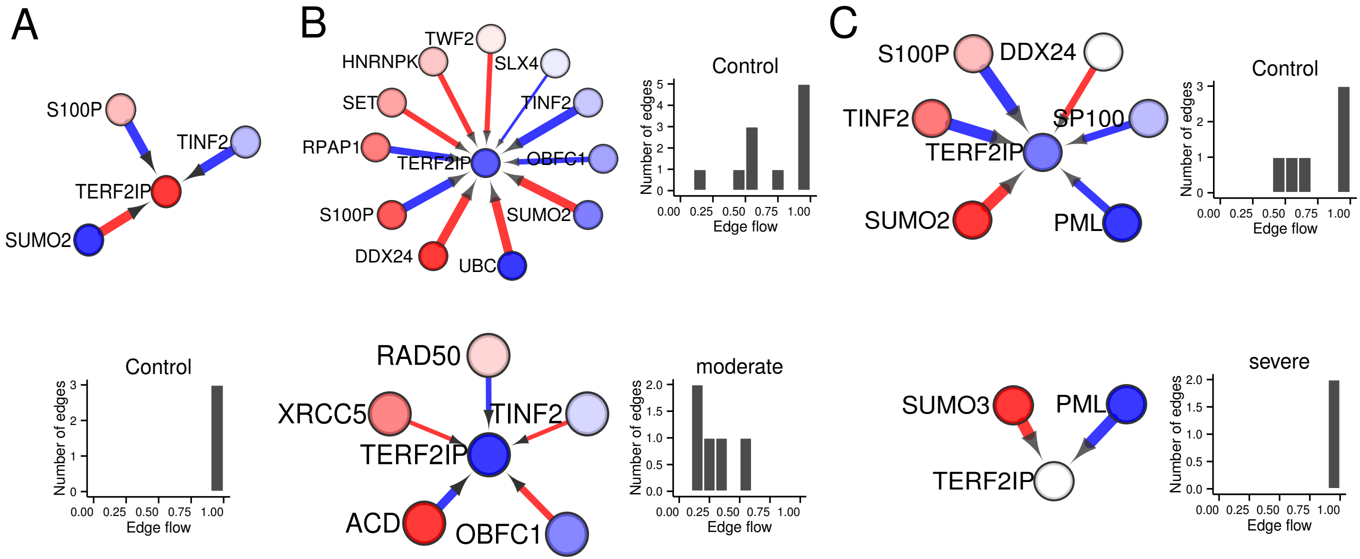

A
S100P
TINF2
TERF2IP
SUMO2
Control
Number of edges
Edge flow
B
TWF2
HNRNPK
SLX4
SET
TINF2
RPAP1
OBFC1
TERF2IP
S100P
SUMO2
DDX24
UBC
Control
Number of edges
Edge flow
RAD50
XRCC5
TINF2
TERF2IP
ACD
OBFC1
moderate
Number of edges
Edge flow
C
S100P
DDX24
TINF2
SP100
TERF2IP
SUMO2
PML
Control
Number of edges
Edge flow
SUMO3
PML
TERF2IP
severe
Number of edges
Edge flow

Figure S13

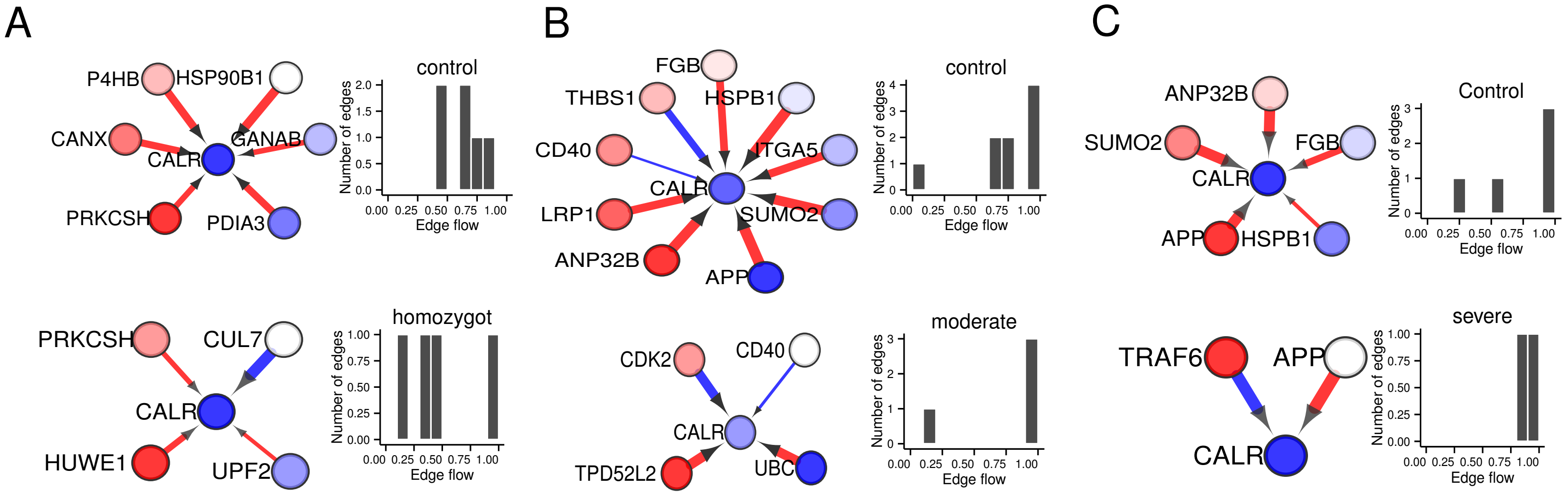
A
P4HB
HSP90B1
CANX
GANAB
CALR
PRKCSH
PDIA3
control
Number of edges
Edge flow
homozygot
PRKCSH
CUL7
CALR
HUWE1
UPF2
B
FGB
THBS1
HSPB1
CD40
ITGA5
CALR
LRP1
SUMO2
ANP32B
APP
control
moderate
CDK2
CD40
CALR
TPD52L2
UBC
C
ANP32B
SUMO2
FGB
CALR
APP
HSPB1
Control
severe
TRAF6
APP
CALR

Figure S14

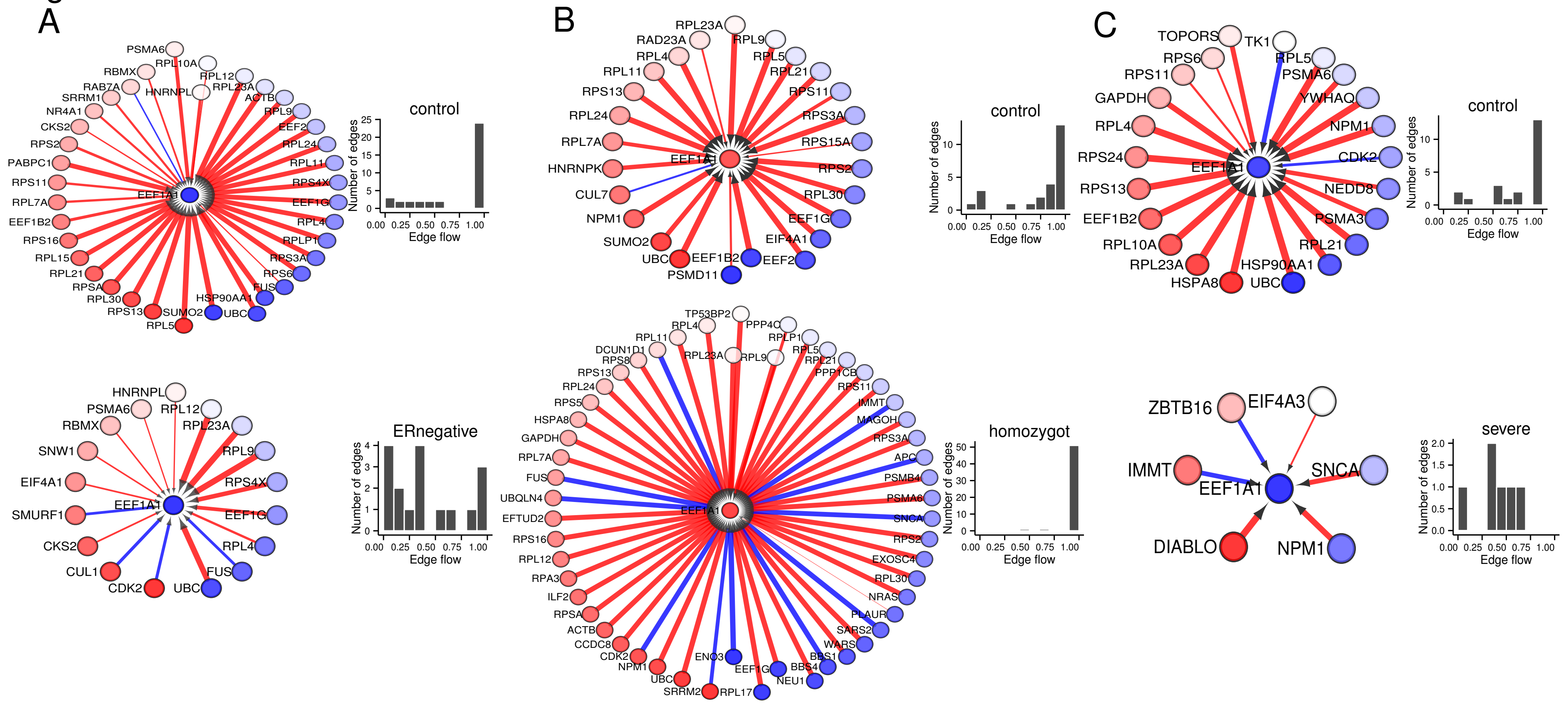
A
B
C
control
ERnegative
homozygot
severe
Edge flow
Number of edges
EEF1A1

Figure S15

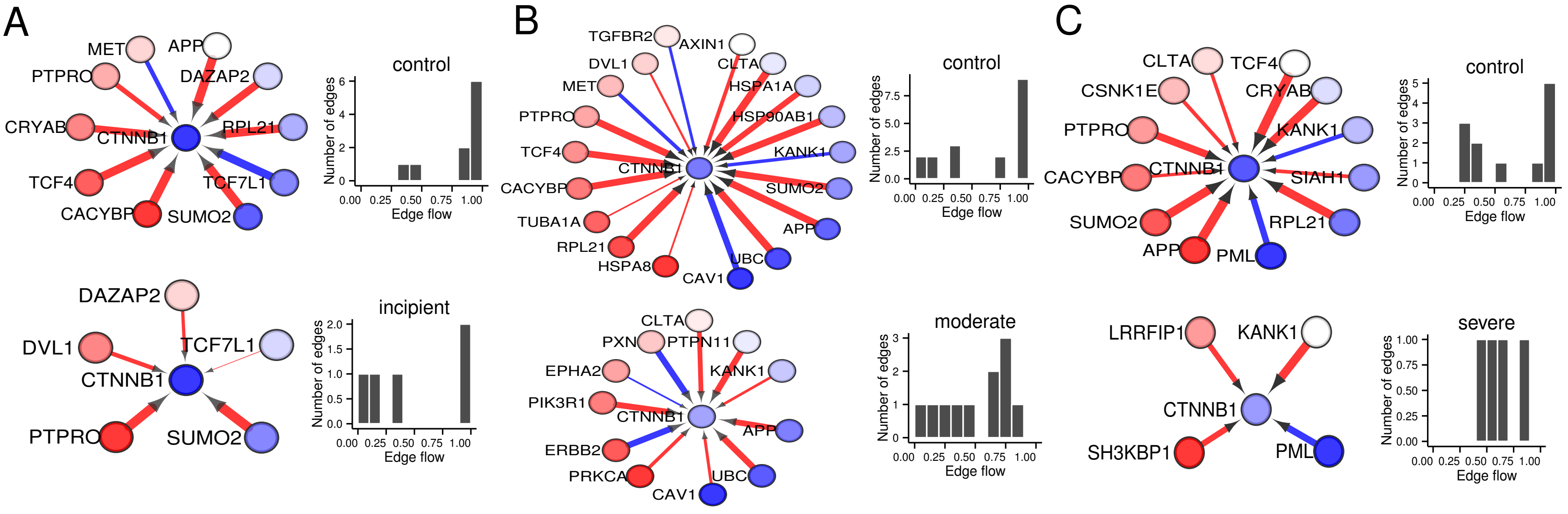
A
MET
APP
PTPRO
DAZAP2
CRYAB
RPL21
CTNNB1
TCF4
TCF7L1
CACYBP
SUMO2
control
Number of edges
Edge flow
DAZAP2
DVL1
TCF7L1
CTNNB1
PTPRO
SUMO2
incipient
Number of edges
Edge flow
B
TGFBR2
AXIN1
DVL1
CLTA
MET
HSPA1A
PTPRO
HSP90AB1
TCF4
KANK1
CTNNB1
CACYBP
SUMO2
TUBA1A
APP
RPL21
UBC
HSPA8
CAV1
control
Number of edges
Edge flow
CLTA
PXN
PTPN11
EPHA2
KANK1
PIK3R1
CTNNB1
APP
ERBB2
PRKCA
UBC
CAV1
moderate
Number of edges
Edge flow
C
CLTA
TCF4
CSNK1E
CRYAB
PTPRO
KANK1
CACYBP
CTNNB1
SIAH1
SUMO2
RPL21
APP
PML
control
Number of edges
Edge flow
LRRFIP1
KANK1
CTNNB1
SH3KBP1
PML
severe
Number of edges
Edge flow

Figure S16

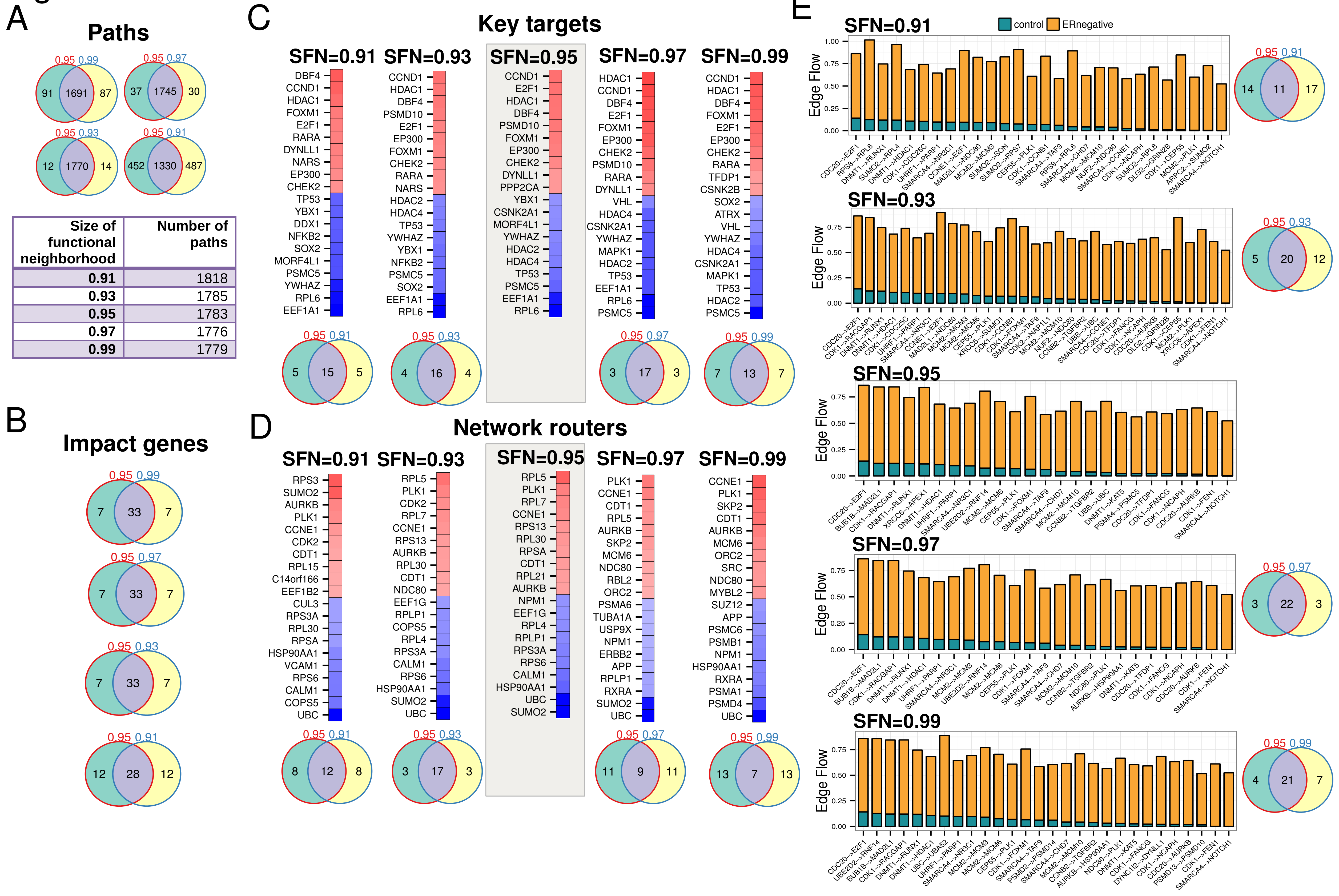

Size of functional neighborhood	Number of paths
**0.91**	1818
**0.93**	1785
**0.95**	1783
**0.97**	1776
**0.99**	1779

Figure S17

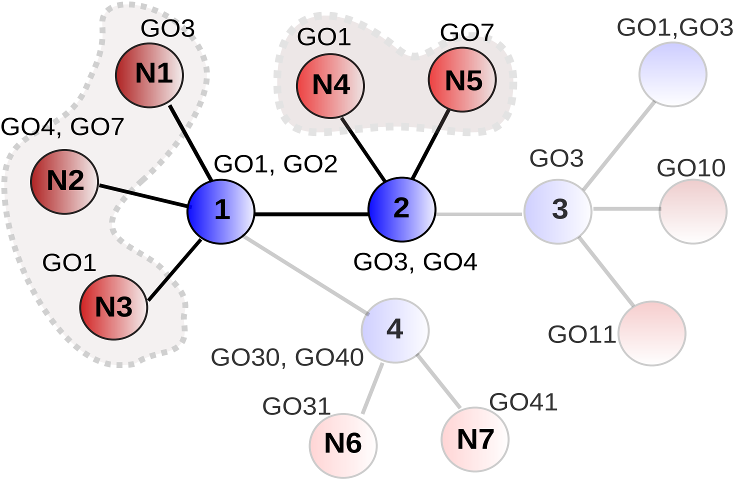

GO3
N1
GO4, GO7
N2
GO1
N3
GO1, GO2
1
GO1
N4
GO7
N5
2
GO3, GO4
GO3
3
GO1,GO3
GO10
GO11
4
GO30, GO40
GO31
N6
GO41
N7

Figure S18

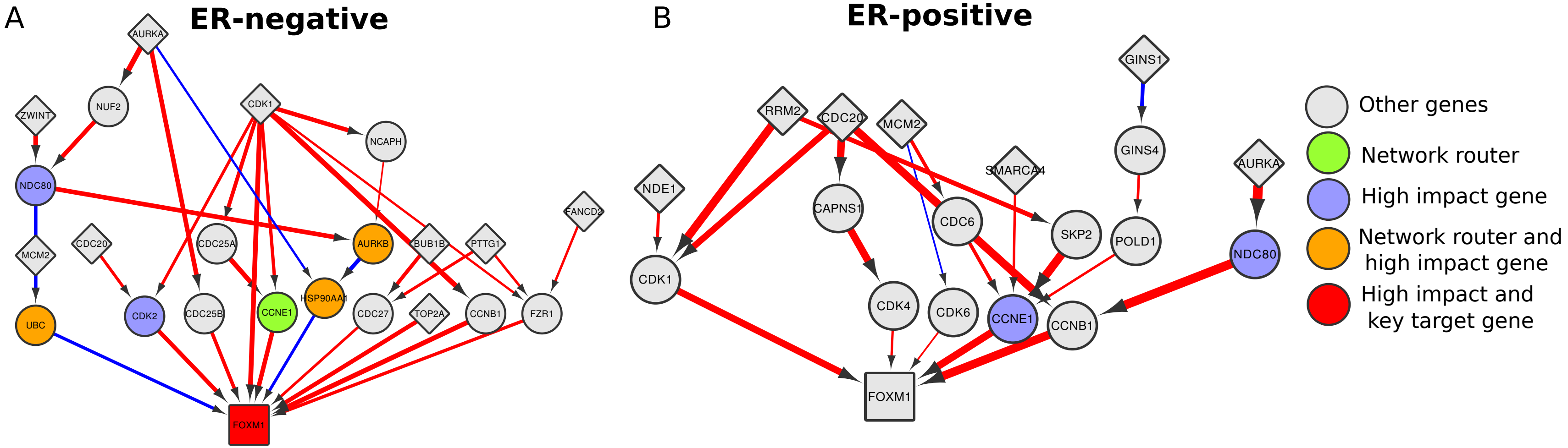
A
ER-negative
AURKA
NUF2
ZWINT
CDK1
NCAPH
NDC80
FANCD2
MCM2
CDC20
CDC25A
AURKB
BUB1B
PTTG1
UBC
CDK2
CDC25B
CCNE1
HSP90AA1
CDC27
TOP2A
CCNB1
FZR1
FOXM1
B
ER-positive
GINS1
RRM2
CDC20
MCM2
GINS4
NDE1
SMARCA4
AURKA
CAPNS1
CDC6
SKP2
POLD1
CDK1
NDC80
CDK4
CDK6
CCNE1
CCNB1
FOXM1
Other genes
Network router
High impact gene
Network router and high impact gene
High impact and key target gene
