# Supplementary material for: NetDecoder: a network biology platform that decodes context-specific biological networks and gene activities
Source: Nucleic Acids Res. 2016 Mar 14;44(10):e100. doi: 10.1093/nar/gkw166 (PMC4889937; doi:10.1093/nar/gkw166)
Supplement: SUPPLEMENTARY DATA [file supp_44_10_e100__index.html]

NetDecoder: a network biology platform that decodes context-specific biological networks and gene activities — NetDecoder: a network biology platform that decodes context-specific biological networks and gene activities — SUPPLEMENTARY DATA 

# NetDecoder: a network biology platform that decodes context-specific biological networks and gene activities

## SUPPLEMENTARY DATA

- SUPPLEMENTARY DATA
